# A systematic review and meta‐analysis of venous thrombosis risk among users of combined oral contraception

**DOI:** 10.1002/ijgo.12455

**Published:** 2018-02-22

**Authors:** Monica V. Dragoman, Naomi K. Tepper, Rongwei Fu, Kathryn M. Curtis, Roger Chou, Mary E. Gaffield

**Affiliations:** ^1^ Department of Reproductive Health and Research WHO Geneva Switzerland; ^2^ Division of Reproductive Health US Centers for Disease Control and Prevention Atlanta GA USA; ^3^ Department of Medical Informatics and Clinical Epidemiology Oregon Health and Science University Portland OR USA

**Keywords:** Combined oral contraceptives, Hormonal contraception, Meta‐analysis, Risk, Systematic review, Venous thromboembolism

## Abstract

**Background:**

Combined oral contraceptives (COCs) containing various progestogens could be associated with differential risks for venous thromboembolism (VTE).

**Objective:**

To evaluate the comparative risks of VTE associated with the use of low‐dose (less than 50 μg ethinyl estradiol) COCs containing different progestogens.

**Search strategy:**

PubMed and the Cochrane Library were searched from database inception through September 15, 2016, by combining search terms for oral contraception and venous thrombosis.

**Selection criteria:**

Studies reporting VTE risk estimates among healthy users of progestogen‐containing low‐dose COCs were included.

**Data collection and analysis:**

A random‐effects model was used to generate pooled adjusted risk ratios and 95% confidence intervals; subgroup and sensitivity analyses assessed the impact of monophasic‐COC use and study‐level characteristics.

**Main results:**

There were 22 articles included in the analysis. The use of COCs containing cyproterone acetate, desogestrel, drospirenone, or gestodene was associated with a significantly increased risk of VTE compared with the use of levonorgestrel‐containing COCs (pooled risk ratios 1.5–2.0). The analysis restricted to monophasic COC formulations with 30 μg of ethinyl estradiol yielded similar findings. After adjustment for study characteristics, the risk estimates were slightly attenuated.

**Conclusions:**

Compared with the use of levonorgestrel‐containing COCs, the use of COCs containing other progestogens could be associated with a small increase in risk for VTE.

## Abstract 2

Combined oral contraceptives containing certain progestogens could be associated with a small increase in risk for venous thromboembolism compared with those containing levonorgestrel.

## 1 INTRODUCTION

Although venous thromboembolism (VTE) is rare among healthy women of reproductive age (incidence 5–10 events per 10 000 women‐years), combined oral contraceptive (COC) use can increase the risk for VTE, including deep venous thrombosis and pulmonary embolism, compared with nonuse.^1^, ^2^ Nonetheless, the incidence of VTE remains low (8–10 events per 10 000 women‐years of exposure) among COC users, and is much lower than the incidence of VTE during pregnancy and the postpartum period.^3^, ^4^ The effect of COCs on the risk of thrombosis was traditionally thought to be solely related to the effects of estrogen on hemostatic factors. However, studies have indicated that the risk of VTE varies among women using COCs containing different progestogens. Given the popularity and widespread use of COCs, any increase in the relative risk of VTE for particular COC formulations could translate to an excess absolute risk of important magnitude.

The present review was conducted for a consultation held by the WHO to examine the venous and arterial risks of COCs, as part of the process of updating the WHO Medical Eligibility Criteria for Contraceptive Use (WHO MEC)^5^; the review and meta‐analysis have been updated since the WHO consultation to include data published during the interim period. For women who wish to use COCs, the key clinical question is whether certain COC formulations might further increase the risk of VTE above that associated with other formulations. Although several other meta‐analyses^6^, ^7^, ^8^, ^9^ on this question have been conducted, the present meta‐analysis updates previous analyses and compares different formulations with a levonorgestrel user group rather than with a nonuser group. The objective of the present systematic review and meta‐analysis was to estimate the risk for VTE among women using COCs containing different progestogens compared with COCs containing levonorgestrel.

## 2 MATERIALS AND METHODS

In the present systematic review and meta‐analysis, PubMed and the Cochrane Library databases were searched for all articles on the association between COC use and VTE in all languages published from database inception through September 15, 2016, using a combination of search terms for oral contraception and venous thrombosis (Table S1). In addition, the reference lists from identified studies and key review articles were hand‐searched for additional studies.

For the exposure, studies were included that reported results for users of COCs with low‐dose ethinyl estradiol (dose <50 μg) coupled with one of the following progestogens: cyproterone acetate, desogestrel, dienogest, drospirenone, gestodene, norgestimate, or levonorgestrel. Studies were only included if the risk estimates were reported separately by COC formulation (including monophasic formulations that had the same dose of ethinyl estradiol in all active pills and multiphasic formulations that had varying doses of ethinyl estradiol throughout the cycle, provided they had the same progestogen). Studies were excluded if COCs containing 50 μg of ethinyl estradiol or more accounted for more than 10% of the total exposure. Also excluded were articles that only reported the risk of VTE among a mixed group of COC users with different progestogen‐containing COCs (e.g., “third generation”), and articles where the reference group cited the use of COC with non‐specified progestogens.^10^, ^11^, ^12^ Five of the included studies did not clearly state the estrogen dose contained; one study^13^, ^14^ spanning 1991 through 1995 relied on the UK Mediplus database, where the majority of recorded prescriptions were for low‐dose COC formulations, and the other four studies^3^, ^15^, ^16^, ^17^ were conducted after 2000, when pills containing 50 μg ethinyl estradiol or more were uncommonly prescribed.

For the outcomes, the present analysis included studies that examined deep venous thrombosis with or without pulmonary embolism; deep venous thrombosis, pulmonary embolism, and venous thrombosis at other sites (cerebral vein, portal vein, caval vein, or renal vein); or unspecified VTE. Studies that only examined pulmonary embolism or fatal VTE were excluded because these are not likely to be representative of the majority of VTE incidents.^18^, ^19^, ^20^ The validation of VTE was factored into the study quality assessment, with VTE cases considered to be validated if they were identified in one of the following ways: (1) from anticoagulation clinics, VTE clinics, or physician report; (2) from discharge diagnosis codes of inpatient hospitalizations; or (3) from diagnosis codes of outpatient records plus additional validation through anticoagulation treatment, medical record review, imaging studies, or physician or patient confirmation. All inpatient VTE diagnoses were considered valid because the diagnostic codes are generally based on confirmed diagnoses. Codes found solely in outpatient data may include codes for both suspected and confirmed diagnoses; therefore, outpatient VTE codes were considered valid only if additional information was examined such as anticoagulation prescriptions, imaging studies, or physician or patient report, in order to exclude suspected VTE that was later ruled out.^21^

Age, personal history of VTE, and recent pregnancy are important risk factors for VTE. Therefore, studies that did not adjust for age were excluded,^22^ and studies were only included if pregnant or postpartum women and women with a history of VTE were excluded from analyses. Exceptions that were included despite these criteria were one study^3^ in which the prevalence of prior VTE was less than 1% among the entire cohort, one study^23^ in which all cases and controls were COC users (because COC use is contraindicated in the context of current or historical VTE^5^), and one study^24^ that excluded women with a recent hospitalization (because this also likely excluded women with a recent pregnancy). A sensitivity analysis was conducted excluding the two studies that did not account for prior VTE^23^ or recent pregnancy^24^ and noted little difference in estimates (data not shown).

When multiple studies were identified that reported results from the same study sample, older analyses^14^, ^25^, ^26^, ^27^ were excluded and only the most recent analyses^23^, ^28^, ^29^, ^30^, ^31^ were included. In addition, nine articles^13^, ^16^, ^23^, ^29^, ^32^, ^33^, ^34^, ^35^, ^36^ reported risk estimates for both cohort and nested case–control analyses for the same study population; in the present analysis, the risk estimates from the nested case–control studies were used because most of the cohort risk estimates were unadjusted. The evidence was summarized and systematically reviewed using standardized abstraction forms. The studies were abstracted by two authors (MVD, NKT) and verified by another (KMC).

Potential sources of bias were assessed for individual studies and quality ratings (good, fair, or poor) were assigned using study‐design‐specific criteria developed by the United States Preventive Services Task Force.^37^ When assessing selection bias in case–control studies, the potential for biased selection of cases and controls (for example, hospital controls vs community controls) and the response rate were considered. The assessment of selection bias in cohort studies involved consideration of whether the cohort represented the population it was taken from, whether the exposed and unexposed groups came from the same population, and whether the follow‐up rate was adequate. The assessment of information bias focused on the determination of contraceptive exposure (for example, self‐report vs pharmacy codes vs medical records) and VTE outcome (for example, diagnostic codes only vs diagnoses objectively confirmed). Finally, it was assessed whether potential confounders were addressed through restriction, matching, or adjustment in analysis; studies that did not control for age, history of VTE, or recent pregnancy were excluded as described above, and other potential risk factors for VTE were considered as potential confounders.

The meta‐analysis included relative risk estimates of VTE that reflected comparisons between pills containing specific progestogen and levonorgestrel formulations. The preferred risk estimates were those with a reference group of users of monophasic COCs containing 30 μg of ethinyl estradiol and levonorgestrel. In some cases, studies reported risk estimates compared with levonorgestrel‐containing COCs as a group but noted that the monophasic preparation represented at least 50% of the total exposure; other studies presented risk estimates compared with any low‐dose levonorgestrel COC. If studies reported multiple risk estimates for users of levonorgestrel‐containing COCs, the risk estimates for the most specific formulations were chosen. In cases where nonusers were the reference group and risk estimates for levonorgestrel and other progestogen‐containing COCs were available, risk estimates with levonorgestrel as the reference group were calculated for inclusion in the meta‐analysis. When studies presented risk ratios for multiple COCs containing the same progestogen at a specific ethinyl estradiol dose, a combined risk ratio for that progestogen was calculated.

A random‐effects model based on profile likelihoods was used to calculate pooled risk ratios.^38^ The presence of statistical heterogeneity was assessed using the standard Cochran χ^2^ test, and the magnitude of the heterogeneity was evaluated using the *I* ^2^ statistic.^39^ The included studies reported different risk estimate measures (odds ratios, hazard ratios, relative risks, or rate ratios). Because VTE is very rare, all these measures provide similar estimates and were combined in a single meta‐analysis.

The analyses were stratified by the specific progestogen formulations. For studies that reported multiple adjusted relative risk estimates, the maximally adjusted estimates were used in the primary analysis. Sensitivity and subgroup analyses were conducted based on whether the study adjusted for body mass index, smoking, or duration of COC use (or assessed these variables as potential confounders and determined adjustment was not needed^3^); the study design (case–control or cohort); the study quality; and the funding source (pharmaceutical industry or other). In addition, a subgroup analysis on users of monophasic COCs containing a standard dose of 30 μg of ethinyl estradiol was conducted to isolate any effect of the progestogen. For comparisons with at least 10 studies, funnel plots and the Egger linear regression method were used to test for small‐study effects (a marker of potential publication bias).^40^ All analyses were performed using Stata/IC version 13.1 (StataCorp, College Station, TX, USA). *P*<0.05 was considered statistically significant.

## 3 RESULTS

**Figure 1:**
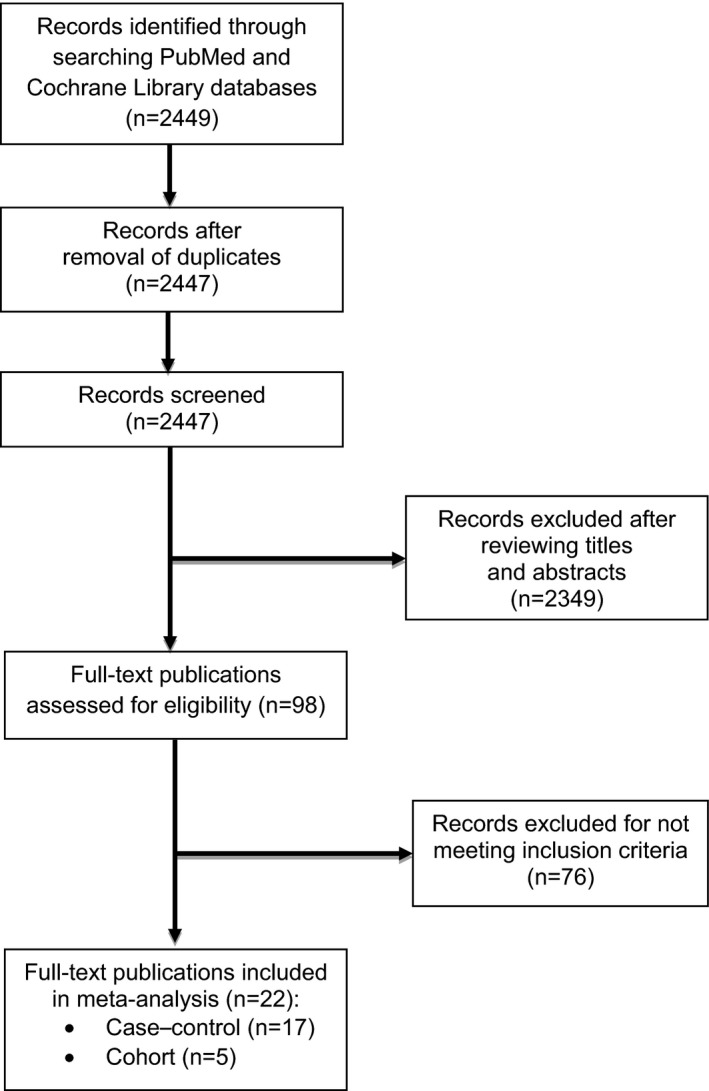
Flow diagram of publication selection for inclusion.

The search strategy identified 2447 unique citations (Fig. 1). Following the evaluation of titles and abstracts and reference lists from key review articles, the full texts of 98 studies were reviewed. Twenty‐two articles satisfied the review inclusion criteria: 17 case–control studies^13^, ^15^, ^16^, ^23^, ^29^, ^30^, ^32^, ^33^, ^34^, ^35^, ^36^, ^41^, ^42^, ^43^, ^44^, ^45^, ^46^ (Table S2), 10 of which were nested within a cohort study, and five cohort studies^3^, ^17^, ^24^, ^28^, ^31^ (Table S3).

All but one of the studies were conducted in Europe or the USA. The remaining study,^41^ sponsored by the WHO, included populations from Europe, Asia, Latin America, and the Caribbean. Most studies, of poor to good quality, retrospectively evaluated large administrative databases. Two cohort studies^3^, ^28^ collected prospective population‐based data with a specific study design; these studies were rated as being of “good” quality (Table S3). All case–control studies and one cohort study^31^ offered evidence of “fair” quality, and the remaining two cohort studies were considered to be of “poor” quality (Tables S2 and S3). Flaws in the studies included the reliance on self‐reported COC use, which could introduce recall bias, or on prescription information, which may not reflect actual use. Additional flaws included small numbers of outcomes and no adjustment for certain risk factors such as the body mass index or smoking.

The use of low‐dose COCs containing cyproterone (nine studies,^17^, ^23^, ^29^, ^30^, ^31^, ^36^, ^41^, ^45^, ^46^ Fig. S1), desogestrel (16 studies,^13^, ^15^, ^17^, ^23^, ^24^, ^29^, ^30^, ^31^, ^32^, ^33^, ^34^, ^41^, ^42^, ^43^, ^45^, ^46^ Fig. S2), drospirenone (10 studies,^3^, ^15^, ^16^, ^17^, ^28^, ^31^, ^35^, ^44^, ^45^, ^46^Fig. S3), or gestodene (12 studies,^13^, ^23^, ^24^, ^29^, ^30^, ^31^, ^32^, ^33^, ^41^, ^42^, ^45^, ^46^ Fig. S4) was associated with an increased risk of VTE compared with the use of levonorgestrel‐containing COCs (Table 1). The use of dienogest was not significantly associated with an increased risk of VTE (Fig. S5); however, only two studies^17^, ^44^ reported dienogest use and the estimate was imprecise. The use of norgestimate‐containing COCs was not associated with an increased risk of VTE versus the use of levonorgestrel‐containing COCs (nine studies,^15^, ^17^, ^23^, ^29^, ^30^, ^31^, ^34^, ^45^, ^46^ Fig. S6). The heterogeneity was moderate (*I* ^2^=30%–66%) for all pooled analyses except for norgestimate (*I* ^2^=15%).

**Table 1:** Summary of meta‐analyses, sensitivity analyses, and subgroup analyses for the risk of venous thromboembolism among users of combined oral contraceptives by progestogen type compared with levonorgestrel

Analysis	Cyproterone acetate			Desogestrel			Dienogest			Drospirenone			Gestodene			Norgestimate		
	No. of studies	*I* ^2^, %	RR (95% CI)	No. of studies	*I* ^2^, %	RR (95% CI)	No. of studies	*I* ^2^, %	RR (95% CI)	No. of studies	*I* ^2^, %	RR (95% CI)	No. of studies	*I* ^2^, %	RR (95% CI)	No. of studies	*I* ^2^, %	RR (95% CI)
Overall	n=9	39.9	2.04 (1.55–2.49)	n= 16	30.1	1.83 (1.55–2.13)	n=2	52.6	1.46 (0.57–5.41)	n=10	66.4	1.58 (1.12–2.14)	n=12	46.5	1.67 (1.32–2.10)	n=9	15.1	1.14 (0.94–1.32)
Exclusion of poor‐quality studies	n=8	43.1	2.05 (1.59–2.53)	n=14	29.7	1.80 (1.51–2.08)	n=1	NA	1.10 (0.54–2.25)	n=9	70.1	1.58 (1.10–2.18)	n=11	46.7	1.63 (1.28–2.04)	n=8	12.0	1.13 (0.91–1.31)
Adjustment for BMI																		
Yes	n=6	56.3	1.78 (0.91–2.82)	n=10	49.4	1.70 (1.28–2.22)	n=0	NA	NA	n=7	60.3	1.60 (0.98–2.42)	n=8	61.2	1.59 (1.14–2.18)	n=6	43.7	1.03 (0.64–1.68)
No	n=3	0.0	2.09 (1.57–3.04)	n=6	0.0	1.92 (1.61–2.35)	n=0	NA	NA	n=3	76.7	1.51 (0.84–2.86)	n=4	0.0	1.84 (1.40–2.47)	n=3	0.0	1.16 (0.93–1.49)
Yes vs no comparison *P* value	0.554			0.416			NA			0.929			0.542			0.648		
Adjustment for smoking																		
Yes	n=5	61.8	1.85 (0.95–3.01)	n=8	50.4	1.78 (1.34–2.37)	n=0	NA	NA	n=3	75.9	1.49 (0.58–3.33)	n=7	59.6	1.68 (1.21–2.35)	n=5	45.6	0.98 (0.57–1.53)
No	n=4	0.0	2.05 (1.53–2.84)	n=8	4.5	1.85 (1.55–2.21)	n=0	NA	NA	n=7	66.2	1.60 (1.08–2.35)	n=5	29.9	1.73 (1.18–2.26)	n=4	0.0	1.18 (0.95–1.56)
Yes vs no comparison *P* value	0.752			0.848			NA			0.844			0.909			0.430		
Adjustment for duration of COC use																		
Yes	n=2	1.0	1.94 (1.11–2.86)	n=6	65.1	1.65 (1.10–2.56)	n=0	NA	NA	n=3	82.6	1.07 (0.37–2.71)	n=4	38.3	1.64 (1.05–2.10)	n=3	0.0	1.17 (0.95–1.49)
No	n=7	50.7	2.08 (1.25–2.99)	n=10	0.0	1.98 (1.67–2.32)	n=0	NA	NA	n=7	57.9	1.77 (1.29–2.47)	n=8	54.1	1.78 (1.28–2.54)	n=6	42.4	1.00 (0.61–1.71)
Yes vs no comparison *P* value	0.770			0.313			NA			0.232			0.518			0.537		
Study design																		
Case–control	n=7	51.2	2.04 (1.33–2.84)	n=13	34.9	1.76 (1.44–2.12)	n=0	NA	NA	n=6	47.6	1.96 (1.28–2.49)	n=10	50.5	1.58 (1.20–2.06)	n=7	22.3	1.11 (0.79–1.34)
Cohort	n=2	2.2	2.04 (0.81–3.34)	n=3	23.4	1.99 (1.46–3.88)	n=0	NA	NA	n=4	76.8	1.30 (0.69–2.26)	n=2	26.6	1.96 (1.38–4.05)	n=2	21.4	1.23 (0.83–2.82)
Case–control vs cohort comparison *P* value	0.924			0.406			NA			0.326			0.344			0.486		
Funding from pharmaceutical industry																		
Yes	n=4	29.9	1.55 (1.02–2.35)	n=9	43.2	1.61 (1.26–2.02)	n=0	NA	NA	n=5	77.7	1.20 (0.67–2.01)	n=7	42.6	1.43 (1.06–1.87)	n=6	29.5	1.07 (0.72–1.34)
No	n=4	51.2	2.23 (1.70–4.04)	n=5	0.0	2.11 (1.74–2.56)	n=0	NA	NA	n=4	0.0	2.12 (1.70–2.68)	n=4	8.7	2.09 (1.52–2.82)	n=2	0.0	1.21 (0.86–1.89)
Not specified	n=1	NA	0.66 (0.07–6.05)	n=2	0.0	3.26 (1.02–8.75)	n=0	NA	NA	n=1	NA	1.57 (0.46–5.37)	n=1	NA	3.90 (1.19–12.79)	n=1	NA	3.24 (0.59–17.77)
Yes vs no comparison *P* value	0.154			0.088			NA			0.096			0.114			0.482		
30 μg ethinyl estradiol	n=0	NA	NA	n=7	43.6	1.66 (1.21–2.33)	n=0	NA	NA	n=4	74.2	1.48 (0.83–2.77)	n=5	0.0	1.46 (1.08–2.06)	n=0	NA	NA

Abbreviations: BMI, body mass index; CI, confidence interval; COC, combined oral contraceptive; NA, not applicable; RR, risk ratio.

Because there was evidence of statistical heterogeneity among the individual studies for many of the progestogens, additional analyses were conducted to assess the possible sources of heterogeneity. In analyses restricted to monophasic COCs with a standard dose of 30 μg of ethinyl estradiol, the risk estimates for desogestrel (seven studies^23^, ^24^, ^29^, ^34^, ^42^, ^43^, ^46^), drospirenone (four studies^3^, ^16^, ^28^, ^35^), and gestodene (five studies^23^, ^24^, ^29^, ^42^, ^46^) were slightly attenuated compared with the risk estimates based on all ethinyl estradiol formulations, and although these three progestogens were all associated with an increased risk, the estimated increase for drospirenone was not significant (Table 1). Restriction of the analysis to monophasic COCs containing 30 μg of ethinyl estradiol did not reduce the heterogeneity except for gestodene. No data were available on the comparative risk of 30‐μg ethinyl estradiol monophasic COCs containing cyproterone, dienogest, or norgestimate.

The findings were also generally consistent in other sensitivity and stratified analyses. The exclusion of poor‐quality studies did not impact the pooled estimates of relative risks or reduce the heterogeneity (Table 1). In stratified analyses, the pooled estimates of risk were generally lower in studies that adjusted for the body mass index, smoking, or the duration of use than in studies that did not adjust for these factors (Table 1). The risk estimates were similar when studies were stratified according to the use of a case–control or cohort design; however, with the exception of deosgestrel and norgestimate, there were some differences in heterogeneity in analyses stratified by study design. Pooled estimates based on studies sponsored by the pharmaceutical industry typically indicated lower risks for VTE but more heterogeneity compared with studies not sponsored by the pharmaceutical industry. None of the differences in the stratified analyses were statistically significant (Table 1).

Sufficient data for the evaluation of potential publication bias were available for desogestrel‐ and gestodene‐containing COCs. The funnel plots were symmetric and there was no statistical evidence for small‐study effects (desogestrel: *P*=0.842; gestodene: *P*=0.599; data not shown).

## 4 DISCUSSION

The present meta‐analysis indicated that the use of low‐dose (less than 50 μg of ethinyl estradiol) COCs containing cyproterone acetate, desogestrel, dienogest, drospirenone, or gestodene was associated with an increased risk (range 1.5–2.0) of VTE compared with the use of levonorgestrel‐containing COCs, although the difference was not statistically significant for dienogest. The use of COCs containing norgestimate was not associated with an increased risk of VTE compared with the use of levonorgestrel.

The estimated risks were only slightly attenuated (compared with the overall analysis) when the analyses were restricted to monophasic COCs containing 30 μg ethinyl estradiol and desogestrel, drospirenone, or gestodene compared with levonorgestrel (there were no data on the risk of monophasic COCs containing 30 μg ethinyl estradiol and cyproterone or dienogest). This finding indicates that the progestogen component could have a role in clot formation; however, the effects of progestogens on the clotting system are not well understood. Although progestogens have not been found to directly induce procoagulant effects, they may counteract the procoagulant effects of estrogen to varying degrees^47^; therefore, it could be that some progestogens decrease the risk of VTE associated with ethinyl estradiol more than other progestogens do. Some studies have found progestogens to be associated with increases in the platelet count and platelet aggregation, whereas others have not.^48^ Further research is needed to determine the hemostatic changes associated with different pill formulations, and to evaluate whether these changes translate into clinical differences in the risk of thrombosis.

The present meta‐analysis used adjusted risk estimates to reduce potential confounding. Four other meta‐analyses^6^, ^7^, ^9^, ^49^ have also estimated pooled relative risks for VTE associated with a specific COC formulation compared with a levonorgestrel‐containing formulation, but have used unadjusted estimates (Table 2). Although each used different methods and varied in the individual studies included, the findings are generally similar. Two of the meta‐analyses^7^, ^9^ found no increase in the risk of VTE with norgestimate‐containing COCs compared with levonorgestrel‐containing pills, which is consistent with the present findings. Similarly, the present findings of slight increases in relative risk for desogestrel, drospirenone, and gestodene are consistent with results from the previous meta‐analyses,^6^, ^7^, ^9^, ^49^ which found small but significantly increased (range 1.3–1.9) relative risks associated with these progestogens. The present estimate for cyproterone acetate was slightly higher (risk ratio 2.0), but generally consistent with the estimates from two other analyses (risk ratio 1.6–1.7).^7^, ^9^

**Table 2:** Pooled estimates (95% confidence intervals) of unadjusted risk ratios for venous thromboembolism among users of combined oral contraceptives by progestogen type compared with levonorgestrel in published meta‐analyses.^a^

Meta‐analysis	Cyproterone	Desogestrel	Dienogest	Drospirenone	Gestodene	Norgestimate
Present analysis	2.04 (1.55–2.49)	1.83 (1.55–2.13)	1.46 (0.57–5.41)	1.58 (1.12–2.14)	1.67 (1.32–2.10)	1.14 (0.94–1.32)
Bateson, 2016^49^						
Prospective cohort studies	—	—	—	0.94 (0.75–1.18)	—	—
Retrospective cohort studies	—	—	—	1.82 (1.60–2.06)	—	—
Stegeman, 2013^9^	1.6 (1.1–2.2)	1.8 (1.4–2.2)	—	1.6 (1.2–2.1)	1.5 (1.2–2.0)	1.0 (0.7–1.3)
Martinez, 2012^7^			—			
Risk ratio	—	1.93 (1.31–2.85)	—	1.67 (1.10–2.55)	1.33 (1.08–1.63)	—
Odds ratio	1.65 (1.30–2.11)	1.62 (1.33–1.97)	—	—	1.49 (1.13–1.96)	1.11 (0.84–1.46)
Kemmeren, 2001^6^	—	1.7 (1.2–2.6)	—	—	1.5 (1.2–2.4)	—

a Estimates are given as risk ratios.

The present analysis had limitations. There are no data from randomized controlled trials; thus, the analysis was limited to comparative observational trials of overall fair quality, which could have resulted in biased results. However, given that VTE is very rare among women of reproductive age, no randomized controlled trials have previously been conducted to investigate this association, and appropriately powered trials would likely be extremely resource‐intensive, limiting their feasibility. It was attempted to reduce bias by including only studies that accounted for important VTE risk factors (for example, age, history of VTE, and recent pregnancy) and by including the maximally adjusted risk estimates in the present calculations; in addition, the findings were similar when poor‐quality studies were excluded from the analysis. Statistical heterogeneity was present in most analyses. Despite the presence of heterogeneity, the findings were generally robust in the subgroup and sensitivity analyses. Most of the exposure and outcome information came from large administrative databases. Although these databases offer greater assurance for capturing specific formulations and duration of use compared with self‐report, prescription data may not accurately represent actual COC use at the time of the VTE event.^50^, ^51^ In addition, the accuracy of administrative databases for the ascertainment of medical conditions such as VTE is variable; however, linking data from these databases to other sources (for example, physician report, evidence for anticoagulation treatment) to verify information reduces the likelihood for misclassification.

In conclusion, the present meta‐analysis indicated that COCs containing certain progestins could confer an increased risk of VTE compared with COCs containing levonorgestrel. This finding should be considered in the context of the overall risk of VTE among women of reproductive age. Any small increase in relative risk accounts for a small number of events at the population level. Assuming a risk of 9–10 VTE events per 10 000 women‐years among women using COCs containing levonorgestrel,^3^, ^28^ the present meta‐analysis indicates that women using COCs containing other progestogens may have a 1.5–2.0‐fold increased risk, resulting in an absolute risk of approximately 14–20 VTE events per 10 000 women‐years, or an additional 5–10 events per 10 000 women‐years. Future research should continue to examine the relative risks associated with different formulations, particularly those for which there is limited evidence, and investigate whether the risks are further elevated in the presence of other VTE risk factors, such as certain medical conditions. In addition, studies should attempt to reduce bias by employing a strong methodology to clearly ascertain and define COC exposure and VTE outcomes and by accounting for important VTE risk factors such as age and prior VTE. Evidence‐based guidelines can be used when counseling women about all contraceptive methods, and for certain women with risk factors for VTE the overall risk of COCs may not be tolerable.^5^ According to the WHO MEC,^5^ the absolute differences between COCs with different progestogens are small and recommendations do not differ based on the progestogen type.

## AUTHOR CONTRIBUTIONS

MVD contributed to the conception and planning of the review, conducting the literature search, data retrieval, writing the initial draft of the manuscript, and revising the manuscript. NKT contributed to the conception and planning of the review, data retrieval, writing the initial draft of the manuscript, and revising the manuscript. RF and RC contributed to the conception and planning of the review, data interpretation, statistical analysis, writing the manuscript, and revising the manuscript. KMC contributed to the conception and planning of the review, verification of data retrieval, writing the initial draft of the manuscript, and revising the manuscript. MEG contributed to the conception and planning of the review, writing the initial draft of the manuscript, and revising the manuscript. All authors approved the final manuscript and agreed to be accountable for the accuracy and integrity of the manuscript's content.

## CONFLICTS OF INTEREST

The authors have no conflicts of interest.

## Supporting information


**Figure S1** Risk for venous thromboembolism among users of combined oral contraceptives containing cyproterone versus levonorgestrel. Abbreviations: CI, confidence interval; NR, not reported; WY, woman‐years. *Number of cases/number of woman‐years of follow‐up. **Number of cases/total number of women.

**Figure S2** Risk for venous thromboembolism among users of combined oral contraceptives containing desogestrel versus levonorgestrel. Abbreviations: CI, confidence interval; WY, woman‐years. *Number of cases/number of woman‐years of follow‐up. **Study included a control group with the same year of birth.

**Figure S3** Risk for venous thromboembolism among users of combined oral contraceptives containing drospirenone versus levonorgestrel. Abbreviations: CI, confidence interval; NR, not reported; WY, woman‐years. *Number of cases/number of woman‐years of follow‐up. **Number of cases/total number of women.

**Figure S4** Risk for venous thromboembolism among users of combined oral contraceptives containing gestodene versus levonorgestrel. Abbreviations: CI, confidence interval; NR, not reported; WY, woman‐years. *Number of cases/number of woman‐years of follow‐up. **Number of cases/total number of women.

**Figure S5** Risk for venous thromboembolism among users of combined oral contraceptives containing dienogest versus levonorgestrel. Abbreviations: CI, confidence interval; NR, not reported. * Number of cases/total number of women.

**Figure S6** Risk for venous thromboembolism among users of combined oral contraceptives containing norgestimate versus levonorgestrel. Abbreviations: CI, confidence interval; NR, not reported; WY, woman‐years. *Study included a control group with the same year of birth. **Number of cases/number of woman‐years of follow‐up. ***Number of cases/total number of women.

**Table S1** Search strategy.

**Table S2** Case–control studies reporting the odds of venous thromboembolism among women using combined oral contraceptives with different types of progestogens.

**Table S3** Cohort studies reporting the risk of venous thromboembolism among women using combined oral contraceptives with different types of progestogens.
