# Supplementary figures and images for: A systematic review and meta‐analysis of venous thrombosis risk among users of combined oral contraception
Source: Int J Gynaecol Obstet. 2018 Feb 22;141(3):287–94. doi: 10.1002/ijgo.12455 (PMC5969307; doi:10.1002/ijgo.12455)

## Slide 1
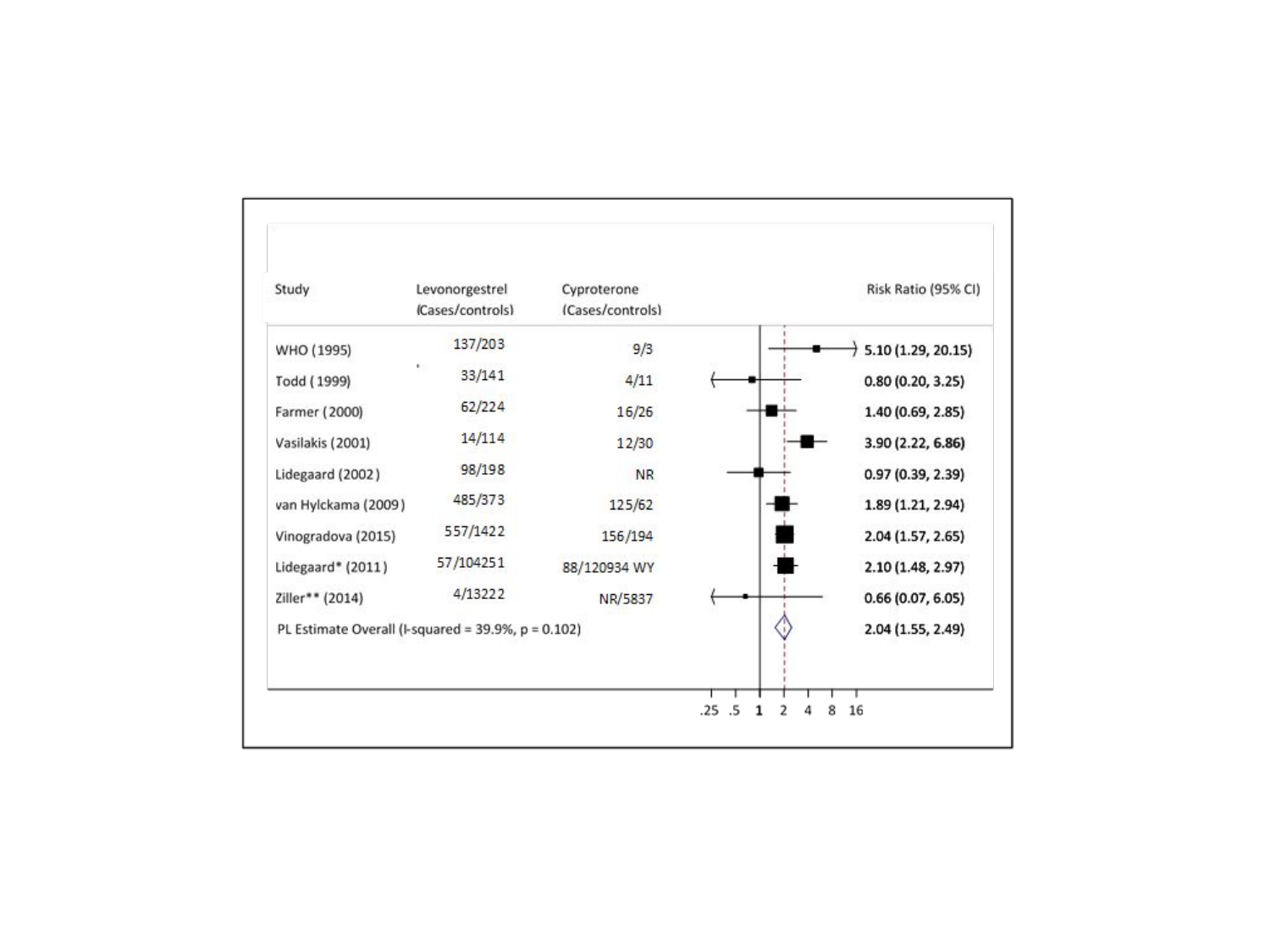

Supplement: Supplementary file 1 — Figure S1 Risk for venous thromboembolism among users of combined oral contraceptives containing cyproterone versus levonorgestrel. Abbreviations: CI, confidence interval; NR, not reported; WY, woman‐years. *Number of cases/number of woman‐years of follow‐up. **Number of cases/total number of women. [file IJGO-141-287-s001.pptx]

## Slide 1
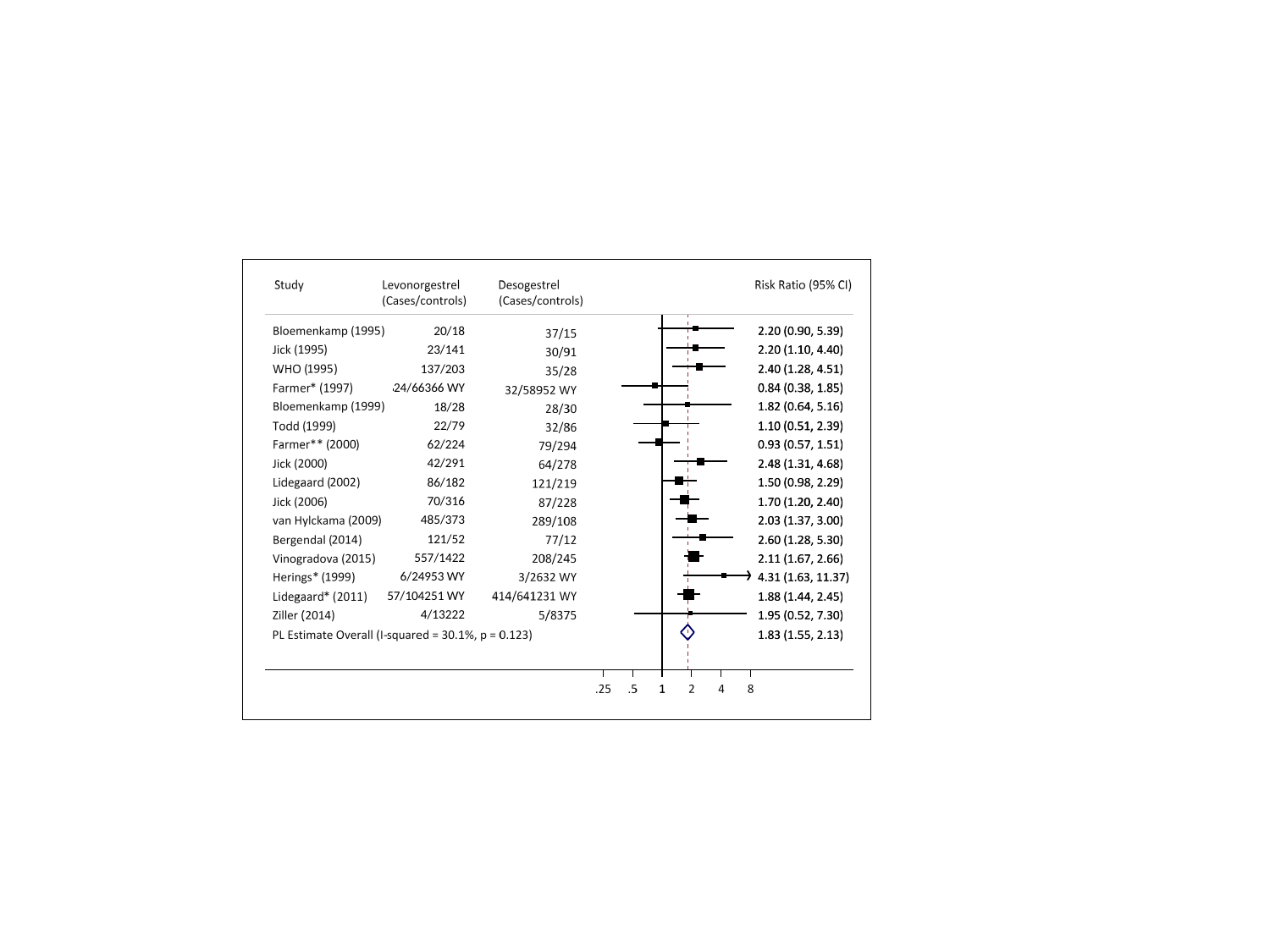

Supplement: Supplementary file 2 — Figure S2 Risk for venous thromboembolism among users of combined oral contraceptives containing desogestrel versus levonorgestrel. Abbreviations: CI, confidence interval; WY, woman‐years. *Number of cases/number of woman‐years of follow‐up. **Study included a control group with the same year of birth. [file IJGO-141-287-s002.pptx]

## Slide 1
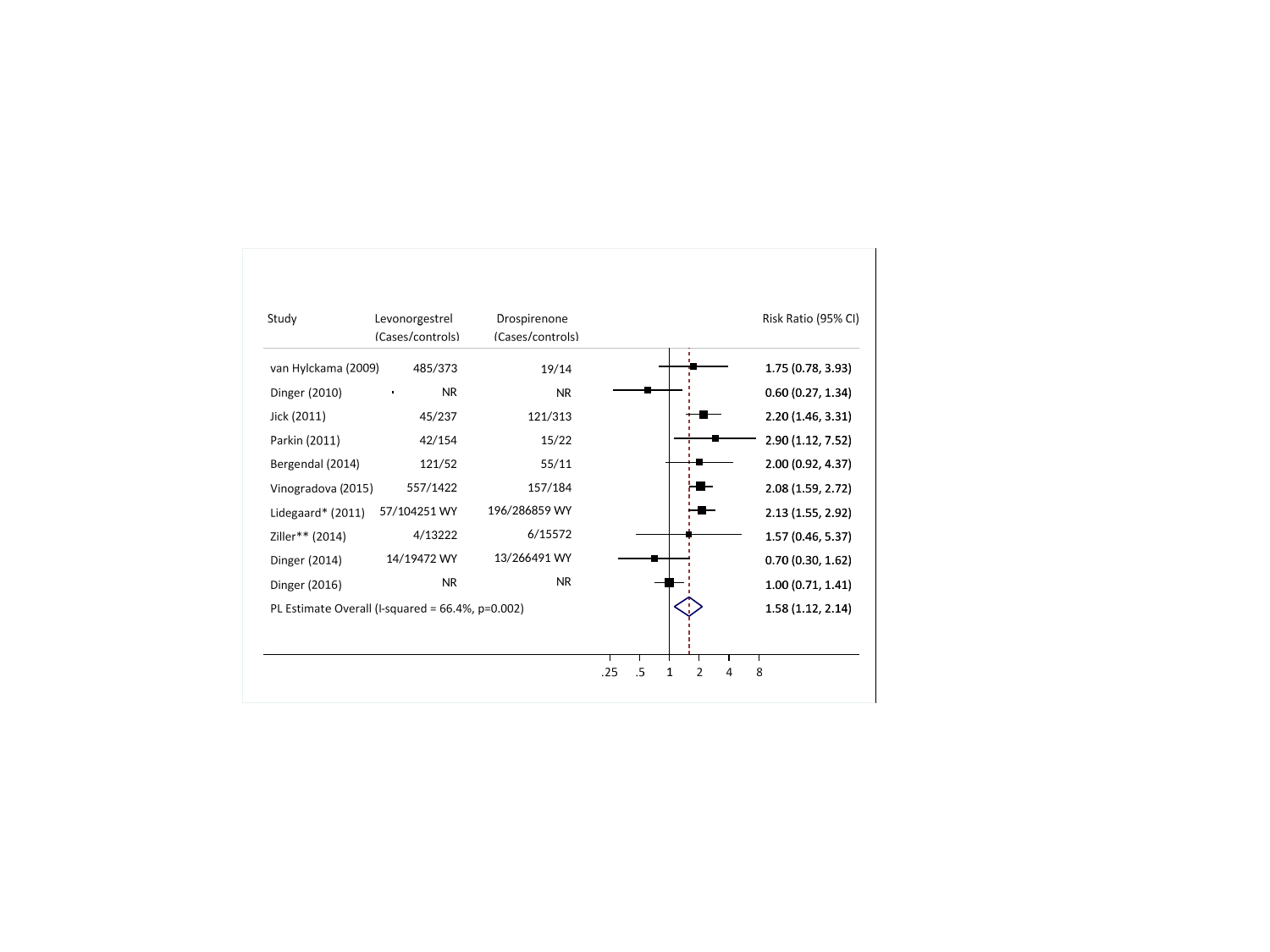

Supplement: Supplementary file 3 — Figure S3 Risk for venous thromboembolism among users of combined oral contraceptives containing drospirenone versus levonorgestrel. Abbreviations: CI, confidence interval; NR, not reported; WY, woman‐years. *Number of cases/number of woman‐years of follow‐up. **Number of cases/total number of women. [file IJGO-141-287-s003.pptx]

## Slide 1
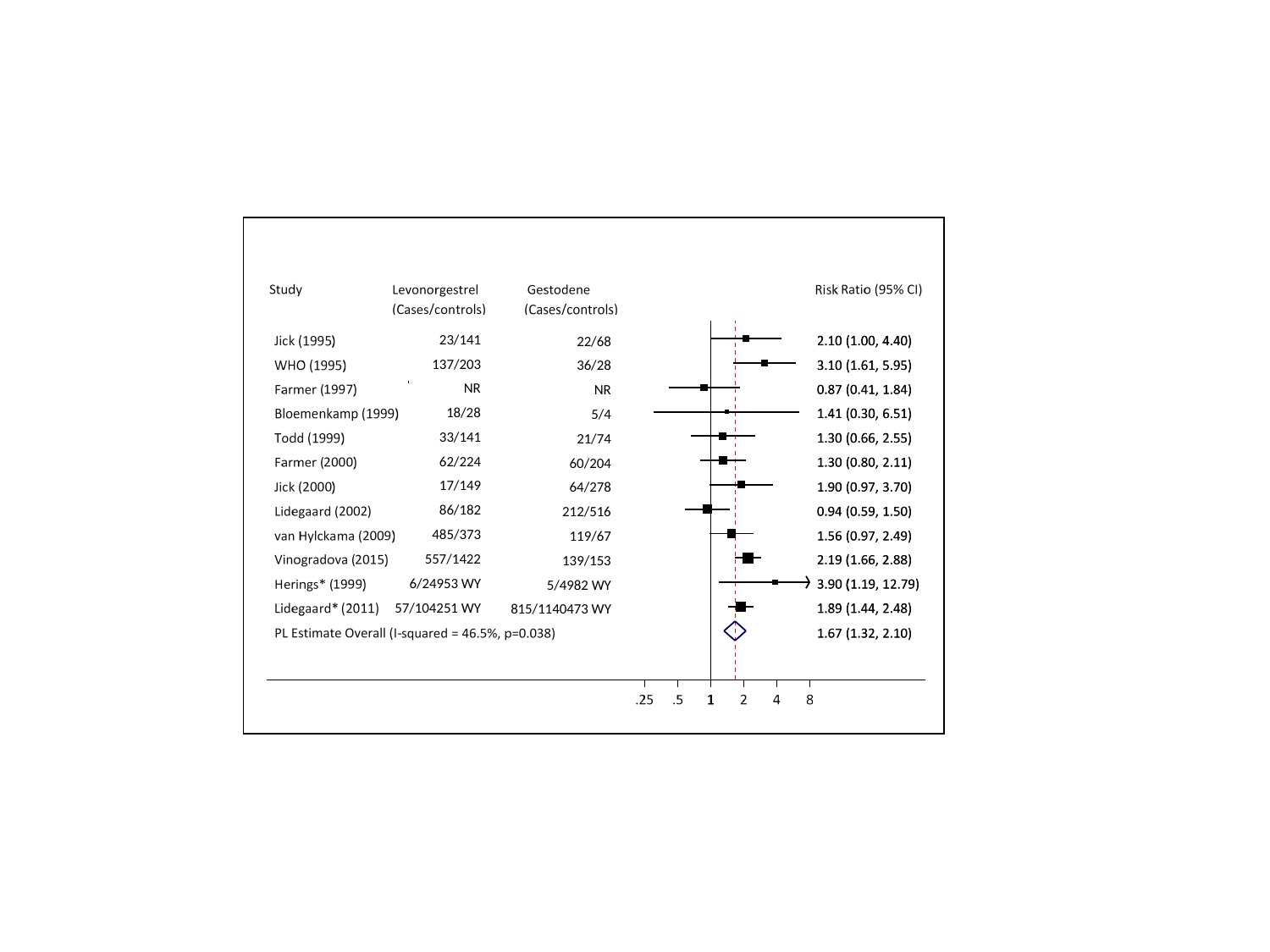

Supplement: Supplementary file 4 — Figure S4 Risk for venous thromboembolism among users of combined oral contraceptives containing gestodene versus levonorgestrel. Abbreviations: CI, confidence interval; NR, not reported; WY, woman‐years. *Number of cases/number of woman‐years of follow‐up. **Number of cases/total number of women. [file IJGO-141-287-s004.pptx]

## Slide 1
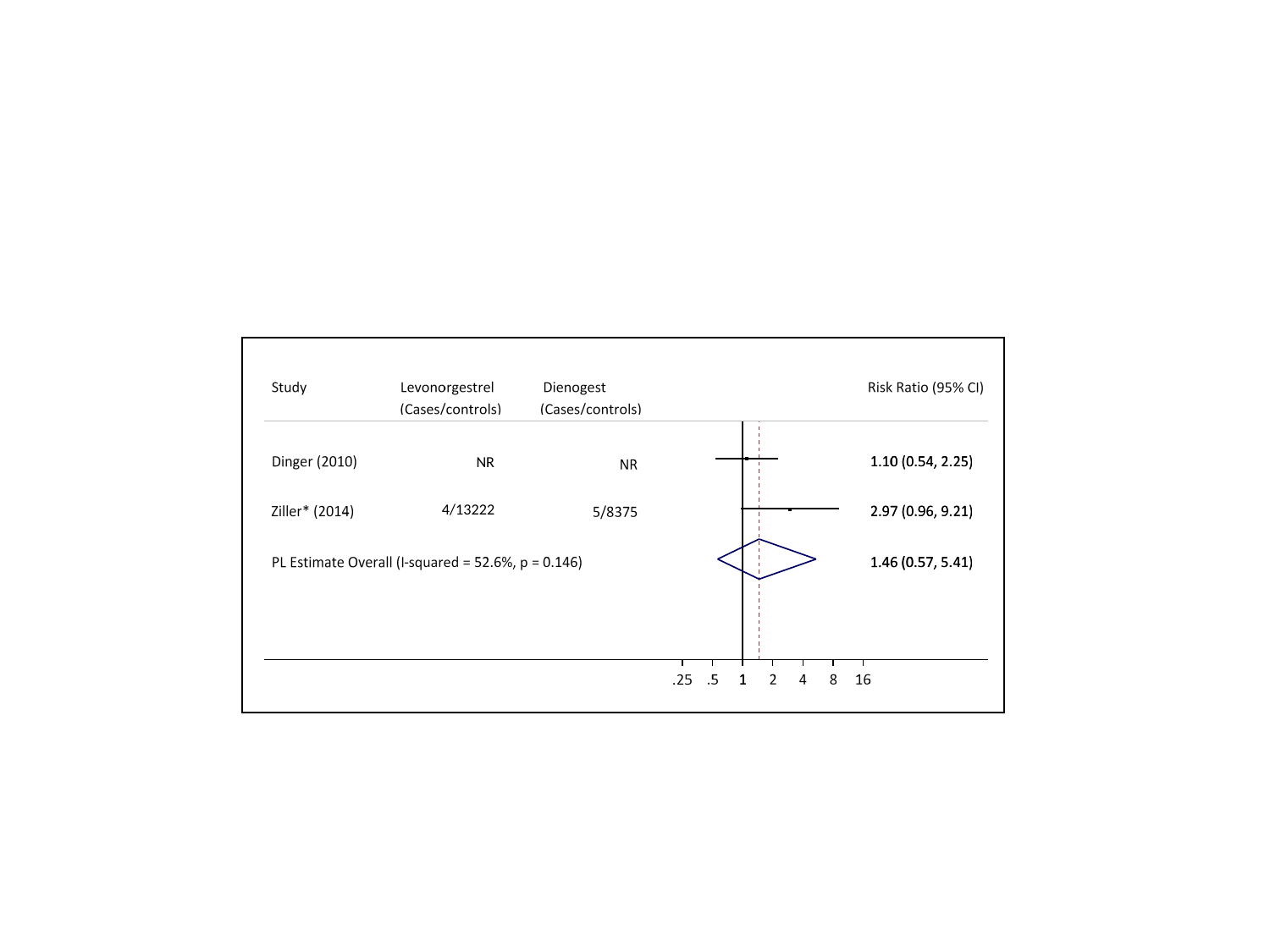

Supplement: Supplementary file 5 — Figure S5 Risk for venous thromboembolism among users of combined oral contraceptives containing dienogest versus levonorgestrel. Abbreviations: CI, confidence interval; NR, not reported. * Number of cases/total number of women. [file IJGO-141-287-s005.pptx]

## Slide 1
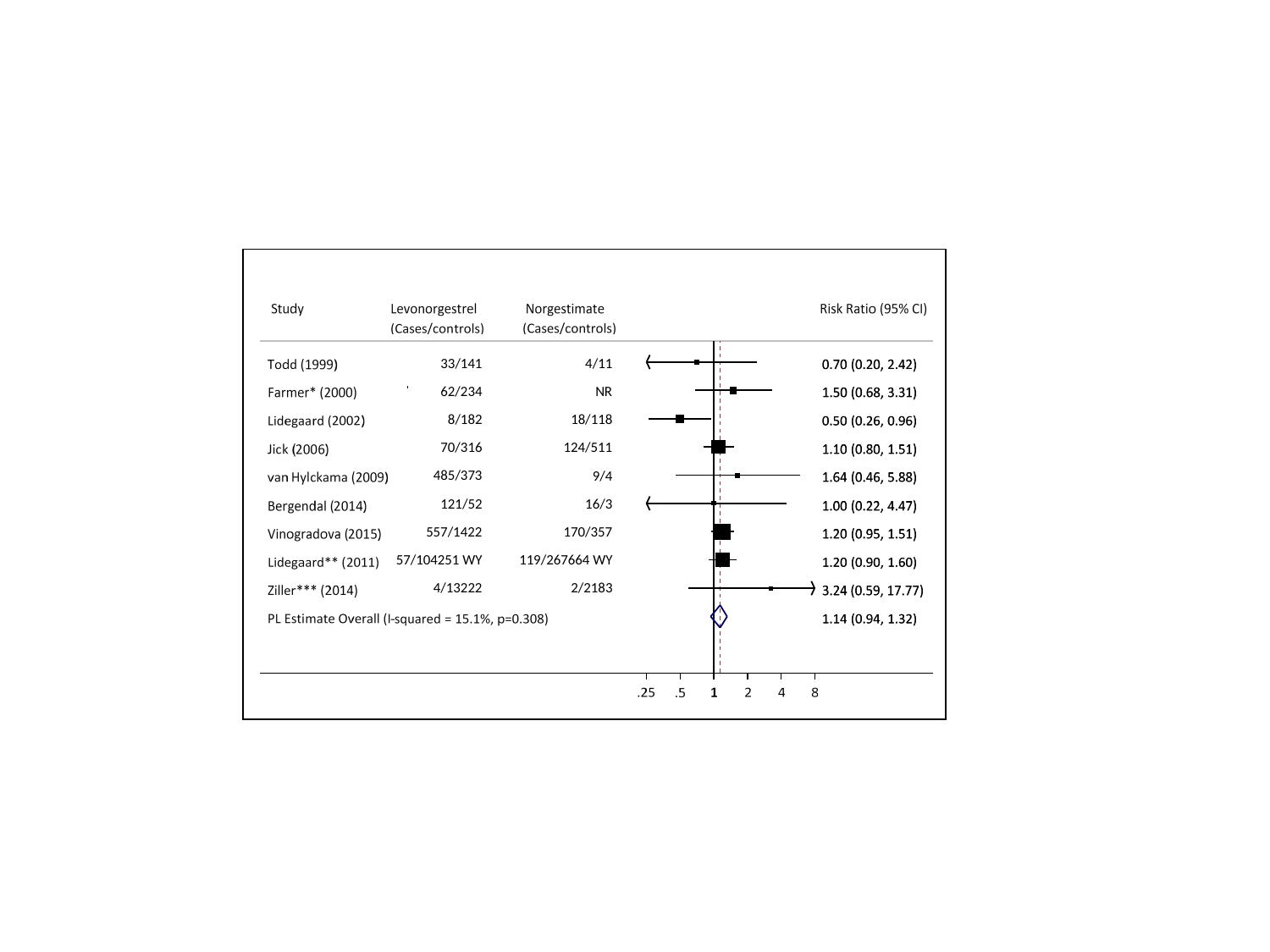

Supplement: Supplementary file 6 — Figure S6 Risk for venous thromboembolism among users of combined oral contraceptives containing norgestimate versus levonorgestrel. Abbreviations: CI, confidence interval; NR, not reported; WY, woman‐years. *Study included a control group with the same year of birth. **Number of cases/number of woman‐years of follow‐up. ***Number of cases/total number of women. [file IJGO-141-287-s006.pptx]
