# Supplementary material for: A systematic review and meta‐analysis of venous thrombosis risk among users of combined oral contraception
Source: Int J Gynaecol Obstet. 2018 Feb 22;141(3):287–94. doi: 10.1002/ijgo.12455 (PMC5969307; doi:10.1002/ijgo.12455)
Supplement: Supplementary file 7 — Table S1 Search strategy. [file IJGO-141-287-s007.docx]

**Table S1** Search strategy

|  | Search terms | Citations |
| --- | --- | --- |
| #1 | "contraceptive agents, female"[MeSH Terms] OR ("contraceptive"[All Fields] AND "agents"[All Fields] AND "female"[All Fields]) OR "female contraceptive agents"[All Fields] OR ("female"[All Fields] AND "contraceptive"[All Fields] AND "agents"[All Fields]) OR "contraceptive agents, female"[Pharmacological Action]) OR ("contraceptives, oral"[MeSH Terms] OR ("contraceptives"[All Fields] AND "oral"[All Fields]) OR "oral contraceptives"[All Fields] OR ("oral"[All Fields] AND "contraceptives"[All Fields]) OR "contraceptives, oral"[Pharmacological Action]) | 71129 |
| #2 | ("venous thrombosis"[MeSH Terms] OR ("venous"[All Fields] AND "thrombosis"[All Fields]) OR "venous thrombosis"[All Fields] OR ("deep"[All Fields] AND "vein"[All Fields] AND "thrombosis"[All Fields]) OR "deep vein thrombosis"[All Fields]) OR DVT[All Fields] OR ("venous thromboembolism"[MeSH Terms] OR ("venous"[All Fields] AND "thromboembolism"[All Fields]) OR "venous thromboembolism"[All Fields]) OR (("veins"[MeSH Terms] OR "veins"[All Fields] OR "venous"[All Fields]) AND ("thromboembolism"[MeSH Terms] OR "thromboembolism"[All Fields] OR ("thromboembolic"[All Fields] AND "event"[All Fields]) OR "thromboembolic event"[All Fields])) OR VTE[All Fields] OR PE[All Fields] OR ("pulmonary"[All Fields] AND "embolus"[All Fields]) OR "pulmonary embolus"[All Fields] | 124213 |
| #3 | #1 AND #2 | 2447 |
