# Supplementary material for: A systematic review and meta‐analysis of venous thrombosis risk among users of combined oral contraception
Source: Int J Gynaecol Obstet. 2018 Feb 22;141(3):287–94. doi: 10.1002/ijgo.12455 (PMC5969307; doi:10.1002/ijgo.12455)
Supplement: Supplementary file 8 — Table S2 Case–control studies reporting the odds of venous thromboembolism among women using combined oral contraceptives with different types of progestogens. [file IJGO-141-287-s008.docx]

**Table S2** Case control studies reporting odds of venous thromboembolism among women using combined oral contraceptives with different types of progestogens

| **Author, Year, Funding, Location** | **Study Design, Study Period** | **Population** | **Ascertainment of Exposure and Outcome** | **COC** | **Results**  **Crude and Adjusted Risk Estimates with co-variates** | **Covariates** | **Strengths** | **Weaknesses** | **Grade** |
| --- | --- | --- | --- | --- | --- | --- | --- | --- | --- |
| Bergendal, 2014 [15]  Janssen-Cilag, Novartis, Organon, Schering, Wyeth, AFA Insurance, Center for Gender Medicine Karolinska Institutet, the Medical Products Agency  Sweden | Case-control  2003-2009 | 948 cases  902 controls  Population-based controls | Exposure: Standardized questionnaires within 90 d of Dx  Main outcome:  DVT (LE or pelvis) and PE  Methods to confirm VTE:  Cases with diagnostic imaging tests and evidence for anticoagulant treatment | LNG  DSG  DRSP  NGM | \| **COC** \| **Cases** \| **Controls** \| **Crude OR (95% CI)** \| **Adjusted OR (95% CI)** \| \| --- \| --- \| --- \| --- \| --- \| \| LNG \| 121 \| 52 \| 1.0 \| 1.0 \| \| DSG \| 77 \| 12 \| 2.8  (1.3-6.0) \| 2.6  (1.3-5.4) \| \| DRSP \| 55 \| 11 \| 2.2  (1.0-5.0) \| 2.0  (0.9-4.3) \| \| NGM \| 16 \| 3 \| 2.3  (0.6-13) \| 1.0  (0.2-4.0) \| | Included women ages 18 to 54 years with first episode of VTE  Excluded women with previous thrombosis, pregnancy in the 3 months before index date or current malignancy (hx of previous malignancy included only if disease free for at least 5 y)  Matching for age (birth year)  Adjusted for BMI, smoking, immobilization | Relied on objective measures for diagnosis of DVT | Recall bias with self-report of COC use; little detail provided regarding rigor with which COC exposure ascertained in baseline questionnaire | II-2, fair |
| Bloemenkamp et al, 1995 [43]  Netherlands Heart Foundation  The Netherlands | Case-control  1988-1992 | 126 cases 159 controls  Population-based controls | Exposure:  Interview- self report, hospital discharge data    Main outcome:  DVT  Methods to confirm VTE:  Cases from anticoagulation clinics | DSG  LNG | \| **COC** \| **Cases** \| **Controls** \| **Adjusted RR (95% CI)** \| \| --- \| --- \| --- \| --- \| \| 30 mcg EE + DSG \| 37 \| 15 \| 8.7 (3.9, 19.3) \| \| 30 mcg EE + LNG \| 20 \| 18 \| 3.8 (1.7, 8.4) \| \| Non-use \| 46 \| 104 \| Ref \| \|  \|  \|  \|  \| \| 30 mcg EE (+ DSG vs. LNG) \| - \| - \| 2.2 (0.9, 5.4) \| | Included women, ages 15 to 49 with first episode of DVT attending one of three anticoagulation clinics  Excluded women with pregnancy, postpartum, postabortion, recent use of injectable progestogens at time of thrombosis or index date (controls)  Matched for age and adjusted for age in reporting RR | COC exposure data supplemented with data from hospital discharge; complete OC data in 95% of users  Relied on objective measures for diagnosis of DVT | Small numbers of cases/controls; overall sample age-matched, but cases more frequently Factor V Leiden +  No information on additional risk factors for VTE (BMI, surgery, prolonged immobilization, recent trauma, smoking status) | II-2, fair |
| Bloemenkamp et al, 1999 [42]  Not stated  The Netherlands | Case-control  1982-1995 | 185 cases 591 controls  Controls from referral population to anticoagulation clinics | Exposure: Questionnaire  Main outcome  DVT  Methods to confirm VTE:  Cases from anticoagulation clinics with clinical evaluation, imaging studies | DSG  GTD  LNG | \| **COC** \| **Cases** \| **Controls** \| **Adjusted OR (95% CI)** \| \| --- \| --- \| --- \| --- \| \| Monophasic,  30 mcg EE+ LNG \| 18 \| 28 \| 3.7 (1.9, 7.2) \| \| Monophasic,  30 mcg EE + DSG \| 22 \| 29 \| 4.9 (2.5, 9.4) \| \| Monophasic,  20 mcg EE + DSG \| 6 \| 1 \| 24.7 (2.8,213.5) \| \| Monophasic,  30 mcg EE + GTD \| 5 \| 4 \| 5.2 (1.3-20.6) \| \| No oral contraceptives \| 83 \| 428 \| Ref \| | Included women ages 15 to 49 seeking care at 2 referral centers for DVT diagnosis  Excluded women without clinical symptoms, VTE other than legs, personal hx of DVT or PE, known inherited clotting defects, pregnant, postpartum, postabortion, cancer, using other sex steroids or other risk factors (IV drug use, nephrotic syndrome), incomplete data on COC use at first visit, or no objective diagnosis with plethysmography and ultrasound    Adjusted for age, family history of VTE, calendar time and center | Relied on objective measures for diagnosis of DVT | Recall bias with self-report of COC use; little detail provided regarding rigor with which COC exposure ascertained in baseline questionnaire  55.1% of cases and 27.5% of controls with report of COC exposure  Complete COC information available for only 70.9% of 265 users  Excluded PE from outcomes/analysis  Did not exclude surgery, trauma, or recent immobilization from this analysis or consider BMI | II-2, fair |
| Dinger et al., 2010 [44]  Bayer Schering Pharma AG  Germany | Case-control  2002-2008 | 680 cases  2720 controls  Population-based controls | Exposure:  Self-administered questionnaire  Main outcome  DVT or PE  Methods to confirm VTE  Letter to physicians, follow up questionnaire to patients, reviewed medical records, confirmed by imaging or less specific diagnostic test or anticoagulant prescription, blind adjudication by MDs  anticoagulation | DNG  DRSP  LNG | All COCs < 30 mcg EE  Idiopathic VTE:   \| **COC** \| **Crude OR (95% CI)** \| **Adjusted OR (95% CI)** \| \| --- \| --- \| --- \| \| DNG/EE \| 1.0 (0.6-2.0) \| 1.1 (0.5-2.1) \| \| DRSP/EE \| 0.7 (0.3-1.4) \| 0.6 (0.3-1.5) \| \| LNG/EE \| Ref \| Ref \|   # of cases and controls not reported | Included women ages 15 to 49 years with clinical diagnosis of VTE  Cases matched to controls according to year of birth and area of residence.  Adjusted for personal history of VTE, family history of VTE, BMI, duration of current COC use. parity, educational level, chronic disease, concomitant medication and smoking  Idiopathic VTE excluded acute risk factors for VTE such as pregnancy, trauma, immobilization, surgery, cancer and chemotherapy | Performed blinded adjudication of all VTE at end of study; 674/680 cases with unanimous decision about VTE diagnosis | Recall bias with self-report of COC use; little detail provided regarding rigor with which COC exposure ascertained in baseline questionnaire | II-2, fair |
| Farmer et al.,  1997[13]  Schering Health Care Ltd and NV Organon  United Kingdom | Nested case-control  1991-1995 | 85 cases  313 controls  Population-based controls | Exposure:  Computer medical records  Main outcome:  VTE unspecified, DVT or PE  Methods to confirm VTE:  Computer diagnosis and anticoagulant prescription, medical record review by MDs  MediPlus database | LNG  DSG  GTD | \| **COC** \| **Adjusted OR**  **(95% CI)** \| \| --- \| --- \| \| GTD vs. All LNG \| 0.87 (0.41-1.83) \| \| DSG vs. All LNG \| 0.84 (0.38-1.85) \| \| 20 mcg EE + DSG  vs. All LNG \| 2.93 (0.86-10.01) \| \| 30mcg EE + DSG vs. All LNG \| 0.64 (0.29-1.45) \| | Included women with VTE diagnosis treated with anticoagulant  Excluded women with personal history of VTE, recent trauma or surgery, pregnant, postpartum, postabortion, use of EC (marker for prescription COC non-use)  Matched cases to controls by exact year of birth, practice site, current use of COC  Adjusted for BMI, change in OC prescribed within 3 months of event, number of prescribed cycles, previous pregnancy, concurrent disease, previous EC use | VTE diagnoses reviewed independently by two physicians, one of whom was an MD familiar with computerized record system  Prescription data may not accurately reflect COC use at time of event; attempted to control for exposure by measuring EC use | Third generation COC users older than second generation users; mean age difference approx. 5 years (25 vs. 20)  Total number of cases and controls assessed and excluded was not reported | II-2, fair |
| Farmer et al., 2000 [23]  NV Organon and Schering AG  United Kingdom | Nested case-control  1992-1997 | 285 cases 1098 controls  Population-based controls (1 analysis matched by practice and year of birth; 1 analysis matched by practice and 5-year age band) | Exposure: Computer records of prescription data  Main outcome:  VTE unspecified, DVT or PE  Methods to confirm VTE:  Computer diagnosis and anticoagulant prescription, computer diagnosis of death from VTE and review of death certificates, questionnaire to some of MDs to confirm event  GPRD database | DSG  GTD  LNG  NGM  CYP | \| **COC** \| **Cases** \| **Controls^a^** \| **Crude OR (95% CI)** \| **Adjusted OR^b^ (95% CI)** \| \| --- \| --- \| --- \| --- \| --- \| \| 30 mcg EE \|  \|  \|  \|  \| \| + LNG \| 62 \| 224 \| Ref \| Ref \| \| +DSG \| 62 \| 218 \| 1.1 (0.7-1.7) \| 1.0 (0.6-1.6) \| \| +GTD \| 60 \| 204 \| 1.1 (0.7-1.7) \| 1.3 (0.8-2.1) \| \| 20 mcg EE \|  \|  \|  \|  \| \| +DSG \| 17 \| 76 \| 0.8(0.4-1.5) \| 0.8 (0.4-1.6) \| \| 35mcg EE \|  \|  \|  \|  \| \| +NGM \| 15 \| 51 \| 1.1(0.6-2.1) \| 1.1(0.6-2.3) \| \| +CYP \| 16 \| 36 \| 1.7 (0.9-3.3) \| 1.4 (0.7-2.9) \|   ^a^ Matched by year of birth  ^b^Analysis of a subset of VTEs verified by MD or death certificate did not substantially change ORs; therefore ORs including all VTEs were included in meta-analysis. | Excluded women with VTE at time of pregnancy or history of delivery, abortion, surgery with general anesthesia, major trauma to lower limbs within 42 days of event; malignancy; concurrent use of other sex hormones with COC; congenital heart disease; events associated with drug overdose; less than 6 months of data available prior to event  Matched by practice and year of birth  Adjusted for BMI, smoking, diastolic blood pressure, asthma, duration of COC exposure, and non-OC/non-asthma prescriptions | Determined case/ control status in GPRD independent of knowledge of OC exposure | Prescription data may not accurately reflect COC use at time of event  Did not account for history of VTE | II-2, fair |
| Jick et al., 1995 [32]  Berlex Labs, Bayer AG, Glaxo Wellcome Inc., Ciba-Geigy Corporation, Hoechst AG, Merck Research Laboratories, Pfizer Inc, Sanofi Winthrop Pharmaceuticals  United Kingdom | Nested case- control  1991-1994 | 75 cases 300 controls  Population-based controls | Exposure:  Computer records of prescription data  Main outcome:  cardiovascular death, nonfatal DVT and/or PE  Methods to confirm VTE:  Deaths from computer report, review of death certificates and necropsy if possible; nonfatal VTE from computer records, questionnaire to MD, review of records by study authors  Excluded cases if no hospital admission, no receipt of anticoagulation, negative diagnostic tests for VTE, pregnant or 3 months postpartum, had recent trauma, or surgery within 3 months  GPRD database | DSG  LNG  GTD | All COCs with < 35 mcg EE; most COCs with EE = 30 mcg; DSG only pill formulation coupled with 20 mcg EE; 5/13 formulations were triphasic (30/40/30)   \| **COC** \| **Cases** \| **Controls** \| **Crude OR (95% CI)** \| **Adjusted OR (95% CI)** \| \| --- \| --- \| --- \| --- \| --- \| \| LNG \| 23 \| 141 \| 1.0 \| 1.0 \| \| DSG \| 30 \| 91 \| 2.3 (1.2-4.6) \| 2.2 (1.1-4.4) \| \| GTD \| 22 \| 68 \| 2.3(1.1-4.8) \| 2.1 (1.0-4.4) \| | Included women 40 years and younger who received one or more prescriptions for OC with < 35mcg EE with LNG, DSG or GTD after Jan 1 1991  Excluded women with history of VTE, stroke, MI, cancer, epilepsy, diabetes, hypertension, hyperlipidemia, cystic fibrosis, pregnant or 3 months postpartum, recent trauma, or recent surgery  Controls matched to cases by age, practice, and index date of case  Adjusted for smoking and BMI | Blinded review of diagnoses for validation  Characteristics of cases and controls similar for age, index year, smoking status, COC formulation, and duration of COC exposure; more cases noted to have BMI >25 (37 vs 18%) | Prescription data may not accurately reflect COC use at time of event | II-2, fair |
| Jick et al., 2000 [33]  AstraZeneca, Berlex Labs, Boehringer Ingelheim Pharmaceuticals,  Boots Healthcare International, Bristol-Myers Squibbs PRI, Glaxo Wellcome, Hoffman-La Roche, Janssen Pharmaceutical Products, RW Johnson PRI, McNeil Consumer Products, Novartis Farmaceutica  UK | Nested case-control  1993-1995  and  1996-1999  (Incidence data from cohort not included in meta-analysis because comparison group contained mixed progestogens) | 106 cases  569 controls | Exposure:  Computer records of prescription data  Main outcome:  VTE (not further specified)  Methods to confirm VTE:  Computer records and anticoagulation prescription, review of computer information by study authors  GPRD database | DSG  GTD  LNG | \| **COC** \| **Crude OR (95% CI)** \| **Adjusted OR^a^ (95% CI)** \| \| --- \| --- \| --- \| \| LNG \| NR \| 1.0 \| \| GTD \| NR \| 1.9 (1.0-3.8) \| \| 20mcg EE + DSG \| NR \| 2.0 (0.9-4.8) \| \| 30mcg EE + DSG \| NR \| 2.8 (1.5-5.3) \|   ^a^Both study periods combined  Authors state that 16 of 71 cases of idiopathic VTE from Study Period 1 included in analysis had been included in their previous report. (53) | Included women, ages 15-39 years, with first VTE diagnosis  Excluded women with < 1 year of data prior to index date, no receipt of anticoagulation, recent injury or surgery to lower limbs within 2 months, currently pregnant or delivery within 3 months, cancer and recent severe trauma  Controls matched on year of birth, practice, COC use on index date  Adjusted for BMI, smoking, duration of use of COCs and switching | Blinded review of VTE diagnosis for validation | Prescription data may not accurately reflect COC use at time of event  No validation of computer records with doctor notes, hospital discharge summaries as in other similar studies  Cases more likely to be obese (43.4% vs. 23.0%) and smokers (38.7% vs. 27.8%) compared to controls though well matched for age, duration of OC use and switching status | II-2, fair |
| Jick  et al.,  2006 [34]  Johnson and Johnson  United States | Nested case-control  2000-2005 | 281 cases 1055 controls  Population-based controls | Exposure:  Drug claims  Main outcome:  DVT or PE  Methods to confirm VTE:  Insurance claims and drug code for anticoagulant prescription  PharMetrics Database | NGM  DSG  LNG | COCs were monophasic and triphasic   \| **COC** \| **Cases** \| **Controls** \| **Crude OR (95% CI)** \| **Adjusted OR (95% CI)** \| \| --- \| --- \| --- \| --- \| --- \| \| 35 mcg EE + NGM \| 124 \| 511 \| 1.1 (0.8-1.5) \| 1.1(0.8-1.5) \| \| 30 mcg EE + DSG \| 87 \| 228 \| 1.7(1.2-2.4) \| 1.7(1.2-2.4) \| \| 30 mcg EE +LNG \| 70 \| 316 \| Ref \| Ref \| | Included women, ages 15 to 39 years, who filled at least one prescription for study medication during study period  Cases: first time recorded diagnosis of VTE with long term (> 6m) anticoagulation  Controls: No VTE with use of OC at index date  Excluded if risk factors for VTE documented in chart within 3 m of index date (trauma to lower limbs, severe trauma, surgery, pregnancy) as well as any history of cancer, renal failure or inflammatory autoimmune disease  Controls matched for age, index date  Adjusted for fibroids, endometriosis, menstrual disorders, hypertension, hyperlipidemia, cardiovascular disease, diabetes, asthma, back pain, recent emergency room visits, recent MD visits | Large database | Exposure: limited to drug claim data, only required one filled prescription; Outcome: limited to diagnostic and procedure codes, no verification of VTE by medical records  Could not adjust for BMI  Only looked back 6 months for contraceptive use, previous history of OC use not known (possible attrition of susceptibles)  Includes only women with insurance through managed care plans | II-2, fair |
| Jick et al., 2011 [16]  No funding  United States | Nested case-control  2002-2008 | 186 cases  681 controls  Population-based controls | Exposure:  Drug claims  Main outcome  DVT or PE  Methods to confirm VTE  Insurance claims and drug code for anticoagulant prescription  Pharmetrics database | DRSP  LNG | \| **COC** \| **Cases** \| **Controls** \| **Crude OR (95% CI)** \| **Adjusted OR (95% CI)** \| \| --- \| --- \| --- \| --- \| --- \| \| *Overall* \|  \|  \|  \|  \| \| LNG \| 65 \| 368 \| Ref \| Ref \| \| DRSP \| 121 \| 313 \| 2.3 (1.6-3.2) \| 2.4 (1.7-3.4) \| \| *20 mcg EE+* \|  \|  \|  \|  \| \| LNG \| 20 \| 131 \| Ref \| Ref \| \| DRSP \| 121 \| 313 \| 2.7 (1.6-4.7) \| 3.2 (1.8-5.5) \| \| *30mcg EE+* \|  \|  \|  \|  \| \| LNG \| 45 \| 237 \| Ref \| Ref \| \| DRSP \| 121 \| 313 \| 2.1 (1.4-3.1) \| 2.2 (1.5-3.4) \| | Included women, ages 15 to 44 years, who filled at least one prescription for study COC during study period  Excluded if risk factors for VTE documented in chart within 3 m of index date (trauma to lower limbs, severe trauma, surgery, pregnancy) as well as any history of cancer, renal failure or inflammatory autoimmune disease  Cases: First time recorded diagnosis of VTE (hospital admission, ER visit or diagnostic test result) with long term (> 6m) anticoagulation  Controls: No VTE with use of OC at index date, matched by year of birth and calendar time  Crude ORs adjusted for age and index year  Adjusted ORs also control for duration of exposure | VTE validated with blinded review to COC exposure  Performed stratified analyses and report no difference between unadjusted and adjusted ORs with obesity and history of menstrual disorder for all women  Analyses restricted to only non-fatal cases of VTE | Cases more likely to be obese (13% vs. 6%)  Among controls, DRSP users younger, shorter duration of use, and new episode of COC use compared to LNG users.  No report on baseline smoking status | II-2, fair |
| Lidegaard et al., 2002 [30]  Organon, Wyeth-Ayerst and Schering AG  Denmark | Case-control  1994-1998 | 987 cases; 4054 controls  Population-based controls | Exposure:  Mailed questionnaire  Main outcome  DVT or PE  Methods to confirm VTE  Hospital discharge diagnosis codes, confirmation from discharging department, patient questionnaires | Estrans LNG  NGM  DSG GTD | \| **COC** \| **Cases** \| **Controls** \| **Crude OR (95% CI)** \| **Adjusted OR (95% CI)** \| \| --- \| --- \| --- \| --- \| --- \| \| LNG \| 98 \| 198 \| 3.2 (2.4-4.3) \| 3.6 (2.6-4.9) \| \| +EE \|  \|  \|  \|  \| \| 50mcg \| 12 \| 16 \| - \| 5.3 (2.3-12.3) \| \| 30-40mcg \| 86 \| 182 \| - \| 3.4 (2.5-4.7) \| \| 20mcg \| 0 \| 0 \| - \| - \| \| POP \| 0 \| 0 \| - \| - \| \| Unspecified \| 0 \| 0 \| - \| - \| \| NGM \| 18 \| 118 \| 1.4 (0.8-2.5) \| 1.7 (1.0-3.2) \| \| +EE \|  \|  \|  \|  \| \| 50mcg \| 0 \| 0 \| - \| - \| \| 30-40mcg \| 18 \| 118 \| - \| 1.7 (1.0-3.2) \| \| 20mcg \| 0 \| 0 \| - \| - \| \| POP \| 0 \| 0 \| - \| - \| \| Unspecified \| 0 \| 0 \| - \| - \| \| DSG \| 121 \| 219 \| 4.2 (3.2-5.6) \| 5.1 (3.8-6.9) \| \| +EE \|  \|  \|  \|  \| \| 50mcg \| 0 \| 0 \| - \| - \| \| 30-40mcg \| 63 \| 90 \| - \| 5.4 (3.6-8.0) \| \| 20mcg \| 58 \| 129 \| - \| 4.8 (3.2-7.1) \| \| POP \| 0 \| 0 \| - \| - \| \| Unspecified \| 0 \| 0 \| - \| - \| \| GTD \| 212 \| 516 \| 3.4 (2.7-4.2) \| 3.5 (2.7-4.4) \| \| +EE \|  \|  \|  \|  \| \| 50mcg \| 0 \| 0 \| - \| - \| \| 30-40mcg \| 206 \| 486 \| - \| 3.5 (2.8-4.5) \| \| 20mcg \| 6 \| 30 \| - \| 2.0 (0.7-5.7) \| \| POP \| 0 \| 0 \| - \| - \| \| Unspecified \| 0 \| 0 \| - \| - \| \| CYP \| NR \| NR \|  \|  \| \| +EE \|  \|  \|  \|  \| \| 30-40mcg \| NR \| NR \| 3.3 (1.4-7.6) \|  \| \| Nonusers (never + former) \| 458 \| 2738 \| Ref \| Ref \|  \| **COC** \| **Corrected OR (95%CI)** \| \| --- \| --- \| \| LNG \| Ref \| \| NGM \| 0.4 (0.2-0.8) \| \| DSG \| 1.6 (1.0-2.4) \| \| GTD \| 1.0 (0.7-1.4) \| \| Nonusers \| Ref \| | Included all women, ages 15 to 44 years  Cases: All women with first episode of VTE  Controls: Between 1994 to 1995, 600 controls derived from age match to women with thrombotic stroke; 96-98, randomly selected from National Patient Register and one-year age matched  Excluded pregnancy  Adjusted for age, year, family VTE, BMI, years of schooling, smoking, diabetes, coagulation disturbances, previous birth  Corrected OR: corrected for duration of use and estrogen dose | Large, population-based database | Cases asked about OC use about one year after event, controls asked about OC use at time of enrolment, may have differential recall  987 cases included in analyses out of 1660 identified; 4054 controls included in analyses out of 4800 identified  Self-reported questionnaire data may have recall bias | II-2, fair |
| Parkin et al., 2011 [35]  No funding  United Kingdom | Nested case-control  2002-2009 | 61 cases  215 controls  Population-based controls | Exposure:  Computer records of prescription data and doctor inquiries  Main outcome:  DVT or PE  Methods to confirm VTE:  Diagnosis codes and anticoagulant prescription, excluded any with continued prescription for OCs after index date, review of computer records by study authors, medical record review of subset of cases  GPRD database | DRSP  LNG | All monophasic COCs containing 30 mcg EE   \| **COC** \| **Cases** \| **Controls** \| **Crude OR (95% CI)** \| **Adjusted OR^a^ (95% CI)** \| \| --- \| --- \| --- \| --- \| --- \| \| *BMI Imputation analysis* \|  \|  \|  \|  \| \| LNG \| 44 \| 189 \| Ref \| Ref \| \| DRSP \| 17 \| 26 \| 3.2 (1.5-7.0) \| 3.3 (1.4-7.6) \| \| *Complete case analysis** \|  \|  \|  \|  \| \| LNG \| 42 \| 154 \| NR \| Ref \| \| DRSP \| 15 \| 22 \| NR \| 2.9 (1.1-7.4) \|     ^a^ Excluded cases and controls missing BMI information; this OR was included in meta-analysis. | Included women, ages 15 to 44 years, with current new use of COC  Excluded women with recorded history of VTE, cancer, chronic renal failure, MI, stroke, other cardiovascular disease, hypertension, hypercholesterolemia, diabetes, colitis, lupus, rheutatoid arthritis, spondylopathies, psoriatic arthritis, cystic fibrosis, injection drug use, coagulation defects, pregnancy within 3 months, surgery, major injury, prolonged immobility, or use of study COC prior to May 2002  Matched controls by year of birth (up to two years), number of years of recorded data, and practice site  Adjusted for BMI as continuous variable with imputation analysis and complete case analysis with available BMI data | Case and control ascertainment independent of COC exposure and verified with record review | Prescription data may not accurately reflect COC use at time of event; considered prescriptions for same episode of COC use if end date for one prescription and issue of new one did not exceed 100 days | II-2, fair |
| Todd et al., 1999 [29]  NV Organon and Schering AG  United Kingdom | Nested case-control  1992-1997 | 99 cases;  366 controls | Exposure: Computer records of prescription data and doctor inquiries  Main outcome:  VTE unspecified, DVT or PE  Methods to confirm VTE:  Computer records plus anticoagulant prescription, recent cases confirmed by questionnaire to MD  Mediplus database | LNG  DSG  GTD  NGM  NRT  CYP | \| **COC** \| **Cases** \| **Controls** \| **Crude OR (95% CI)** \| **Adjusted OR (95% CI)** \| \| --- \| --- \| --- \| --- \| --- \| \| 30 mcg EE \|  \|  \|  \|  \| \| +LNG \| 22 \| 79 \| Ref \| Ref \| \| +DSG \| 23 \| 63 \| 1.5 (0.7-3.1) \| 1.1 (0.5-2.6) \| \| +GTD \| 21 \| 71 \| 1.1 (0.5-2.3) \| 1.1 (0.5-2.4) \| \| 20mcg EE \|  \|  \|  \|  \| \| +DSG \| 9 \| 23 \| 1.3 (0.4-3.6) \| 1.1 (0.4-3.4) \| | Included women, ages 15 to 49, treated with anticoagulation and exposed to COC  Excluded women with prior VTE, pregnant or within 6 weeks postpartum, or abortion, surgery with general anesthesia, major trauma to lower limbs, use of concurrent sex hormones, or malignant disease within 6 weeks of VTE and at least 6 months of record data prior to VTE  Controls matched by practice site and year of birth  Adjusted for BMI, smoking, diastolic blood pressure, number of non-OC scripts | Blinded assessment of VTE diagnosis (57/99 cases) | Don't state how many overall cases ages 18-50 before exclusions  Prescription data may not accurately reflect COC use at time of event | II-2, fair |
| van Hylckma et al., 2009 [46]  Netherlands Heart Foundation, Dutch Cancer Foundation, Netherlands Organisation for Scientific Research  The Netherlands | Case-control  1999-2004 | 1524 cases; 1760 controls  (712 female partner controls; 1048 random digit dialing controls) | Exposure:  Mailed questionnaire, follow up interview by phone or in person  Main outcome  DVT or PE  Methods to confirm VTE  Cases from anticoagulation clinics, review of records from hospital and MD including imaging, patient questionnaire  MEGA study | LNG  GTD DSG NET CYP NGM DRSP | \| **COC** \| **Cases** \| **Controls** \| **Adjusted OR (95% CI)** \| \| --- \| --- \| --- \| --- \| \| GTD^a^ \| 119 \| 67 \| 5.6 (3.7-8.4) \| \| DSG^a^ \| 289 \| 108 \| 7.3 (5.3-10.0) \| \| CYP \| 125 \| 62 \| 6.8 (4.7-10.0) \| \| DRSP \| 19 \| 14 \| 6.3 (2.9-13.7) \| \| LNG* \| 485 \| 373 \| 3.6 (2.9-4.6) \| \| NGM \| 9 \| 4 \| 5.9 (1.7-21.0) \|   ^a^Analysis restricted to preparation with most commonly used dose of estrogen: 30 mcg EE | Included women, ages 18 to 50, with first episode of VTE  Excluded postmenopausal women, pregnant or within 4 weeks postpartum, women using hormonal contraception other than oral, and history VTE  Adjusted for age and inclusion period | Interview data may have recall bias, but questionnaire filled in within few weeks after VTE | Don't state how many overall cases ages 18-50 before exclusions or how many cases/controls in this age group received questionnaires but didn't respond  Did not control for confounders like obesity, smoking, other medical conditions | II-2, fair |
| Vasilakis et al., 2001 [36]  Boston Collaborative Drug Surveillance Program  United Kingdom | Nested case-control  1992-1999 | Study population: 24,401 women using cyproterone 75,000 women using levonorgestrel; Analyses: 26 cases 144 controls | Exposure:  Prescription database, at least 1 prescription for study COC  Main outcome:  VTE (not further specified)  Methods to confirm VTE:  Computer diagnoses of hospitalized cases plus anticoagulant prescription or positive imaging, review of records from MD  GPRD | CYP  LNG | All COCs reported to be “low-dose”   \| **COC** \| **Cases** \| **Controls** \| **Crude OR (95% CI)** \| **Adjusted OR (95% CI)** \| \| --- \| --- \| --- \| --- \| --- \| \| CYP \| 12 \| 30 \| 3.2 (1.4-7.6) \| 3.9 (1.1-13.4) \| \| LNG \| 14 \| 114 \| Ref \| Ref \| | Included women, ages 16 to 39 years, with at least one prescription and first-time episode of idiopathic VTE  Controls matched for age within 1 year  Excluded from analysis women with a history of recent immobilization, surgery, pregnancy or trauma and anyone without at least one year of recorded medial data  Adjusted for BMI, smoking, hirsutism, acne, PCOS, asthma  Reports duration of exposure did not materially affect risk estimates |  | Prescription data may not accurately reflect COC use at time of event  Small number of cases, wide confidence intervals  Did not assess past use of COCs; Not able to verify all cases with medical record review | II-2, fair |
| Vinogradova et al., 2015 [45]  No external funding  United Kingdom | Nested case-control  2001-2013 [52] | CPRD  5062 cases  19638 controls  QResearch  5500 cases  22396 controls  Controls matched on age, practice and calendar year  2001-2013 | Exposure:  Prescription data  Outcome:  Clinical code in GP record, linked hospital record or linked mortality record  CPRD (only GP records)  Methods to confirm VTE: ICD-10 codes from GP practice linked to hospital admission in Q Research; conducted sub-analyses for both databases among women treated with anticoagulants, linking ICD-10 codes from GP to treatment | CYP  DRSP  DSG  GTD  NGM  LNG | All COCs with 30-40 mcg EE   \| **COC** \| **Cases** \| **Controls** \| **Adjusted OR (95% CI)** \| \| --- \| --- \| --- \| --- \| \| CPRD \| \| \| \| \| CYP \| 83 \| 99 \| 2.00 (1.38-2.89) \| \| DRSP \| 94 \| 108 \| 2.14 (1.49-3.06) \| \| DSG \| 113 \| 113 \| 2.61 (1.87-3.65) \| \| GTD \| 57 \| 61 \| 2.44 (1.58-3.77) \| \| NGM \| 71 \| 181 \| 0.89 (0.64-1.26) \| \| LNG \| 260 \| 683 \| 1.00 \| \| QResearch \| \| \| \| \| CYP \| 73 \| 95 \| 2.08 (1.43-3.03) \| \| DRSP \| 63 \| 76 \| 2.02 (1.35-3.01) \| \| DSG \| 95 \| 132 \| 1.74 (1.26-2.41) \| \| GTD \| 82 \| 92 \| 2.03 (1.42-2.90) \| \| NGM \| 99 \| 176 \| 1.53 (1.12-2.09) \| \| LNG \| 297 \| 739 \| 1.0 \| \| Combined analysis \| \| \| \| \| CYP \|  \|  \| 2.04 (1.57-2.65) \| \| DRSP \|  \|  \| 2.08 (1.59-2.72) \| \| DSG \|  \|  \| 2.11 (1.68-2.67) \| \| GTD \|  \|  \| 2.19 (1.66-2.88) \| \| NGM \|  \|  \| 1.20 (0.95-1.51) \| \| LNG \|  \|  \| 1.0 \| | Included women, ages 14 to 49 years with at least one prescription and first-time episode of VTE  Controls matched by practice site and year of birth  Excluded women with prescriptions for anticoagulant more than 6 weeks before index date among cases and any AC prescription before the index date for controls; women with hysterectomy, oophorectomy, sterilization; and, women with pregnancy or within three months after delivery.  Adjusted for BMI, smoking status, alcohol consumption, ethnicity, chronic and acute medical conditions, and use of other hormonal contraceptives | Large, population-based databases | Prescription data may not accurately reflect COC use at time of event  VTE diagnosis limited to diagnostic codes and treatment; one database linked to hospital admissions; no verification of VTE by medical records | II-2, fair |
| WHO, 1995 [41]  UNDP, UNFPA, WHO, World Bank  Brazil  Chile  Colombia  Germany  Hong Kong  Hungary  Jamaica  Thailand  UK | Case-control  1989-1993 | 769 cases  1979 hospital controls, 246 general practice controls in 4 centers  Controls matched on age and hospital or general practice | Exposure:  Questionnaire  Main outcome:  DVT or PE  Methods to confirm VTE:  Monitoring system to identify eligible cases prompting review of medical history, examinations and diagnostic testing by panel of clinical experts | DSG  GTD  CYP  LNG | \| **COC** \| **Cases** \| **Controls** \| **Crude OR (95% CI)** \| \| --- \| --- \| --- \| --- \| \| DSG \| 35 \| 28 \| 2.6 (1.4-4.8) \| \| GTD \| 36 \| 28 \| 2.6 (1.4-4.8) \| \| 35mcg EE  +CYP \| 9 \| 3 \| 5.1 (1.3-20.3) \| \| LNG \| 137 \| 203 \| Ref \|   All COCs < 35 mcg EE   \| **COC** \| **OR adjusted for BMI (95% CI)** \| **OR adjusted for BMI and alcohol (95% CI)** \| \| --- \| --- \| --- \| \| DSG \| 2.2 (1.2-4.1) \| 2.4 (1.3-4.6) \| \| GTD \| 3.0 (1.6-5.8) \| 3.1 (1.6-5.9) \| \| 35mcg EE +  CYP \| NR \| NR \| \| LNG \| Ref \| Ref \| | Included women, ages 15 to 49 (20-44 at three centers)  Excluded deaths within 24 hrs of admission, history stroke, MI, prior DVT or PE, natural or surgical menopause, recent pregnancy, major illness with prolonged bed rest, or major surgery  Controls matched on age and hospital or general practice  Adjusted for BMI, alcohol consumption | VTE diagnoses verified by review of medical records and imaging  Multiple countries and centers | Small numbers of cases and controls for certain progestin types  924 community controls identified as potential, only 491 sent letters, reasons not stated  Non-response rates for community controls high (39% of those sent letters)  Cases had higher mean BMI than controls | II-2, fair |

Abbreviations: BMI, body mass index; CI, confidence interval; COC, combined oral contraceptive; CYP, cyproterone acetate; DNG, dienogest; DRSP, drospirenone; DSG, desogestrel; DVT, deep venous thrombosis; EC, emergency contraception; EE, ethinyl estradiol; GPRD, General Practice Research Database; GTD, gestodene; LNG, levonorgestrel; MI, myocardial infarction; NGM, norgestimate; NR, not reported; OC, oral contraceptive; OR, odds ratio; PCOS, polycystic ovarian syndrome; PE, pulmonary embolism; POP, progestogen-only pill; RR, relative risk; VTE, venous thromboembolism.

**Supplementary reference**

52. Vinogradova Y, Coupland C, Hippisley-Cox J. Exposure to combined oral contraceptives and risk of venous thromboembolism: a protocol for nested case-control studies using the QResearch and the CPRD databases. BMJ open. 2014;4(4):e004499.
