# Supplementary material for: A systematic review and meta‐analysis of venous thrombosis risk among users of combined oral contraception
Source: Int J Gynaecol Obstet. 2018 Feb 22;141(3):287–94. doi: 10.1002/ijgo.12455 (PMC5969307; doi:10.1002/ijgo.12455)
Supplement: Supplementary file 9 — Table S3 Cohort studies reporting the risk of venous thromboembolism among women using combined oral contraceptives with different types of progestogens. [file IJGO-141-287-s009.docx]

**Table S3** Cohort studies reporting risk of venous thromboembolism among women using combined oral contraceptives with different types of progestogens

| **Author, Year, Funding, Location** | **Study Design, Study Period** | **Population** | **Ascertainment of Exposure and Outcome** | **Progestogen** | **Results**  **Crude and Adjusted Risk Estimates with co-variates** | **Covariates** | **Strengths** | **Weaknesses** | **Grade** |
| --- | --- | --- | --- | --- | --- | --- | --- | --- | --- |
| Dinger et al., 2016 [28]  Bayer AG  Austria Belgium Denmark France Germany Netherlands United Kingdom | Prospective cohort  2000-2005 | 59,510 women enrolled 836 (1.4%) excluded 58,674 women analyzed 318,784 WY observation | Exposure:  Questionnaire  Outcome:  Self-administered questionnaire every 6 months; Information from physician to validate VTE for 5 years, then every 12 months through up to 10 years | DRSP  LNG | \| **COC** \| **VTE** \| **Incidence (VTE/**  **10,000 WY** \| **95% CI** \| \| --- \| --- \| --- \| --- \| \| All DRSP \| 56 \| 10.7 \| (8.1-13.9) \| \| DRSP/30 EE \| 56 \| 10.7 \| (8.1-13.9) \| \| All LNG \| 53 \| 9.2 \| (6.9 -12.0) \| \| LNG/30 EE \| 35 \| 10.9 \| NR \| \| LNG/ <30 EE \| 18 \| 7.5 \| NR \|  \|  \| **Adjusted HR**  **(95% CI)** \| \| --- \| --- \| \| *All VTE*  DRSP vs. LNG \| 1.1 (0.8- 1.7) \| \| DRSP/30 EE vs. LNG/30 EE \| 1.0 (0.6 – 1.5) \| \| *Idiopathic VTE*  DRSP vs LNG \| 1.0 (0.6 - 1.2) \| | Adjusted for age, BMI, duration of use, family history of VTE  Baseline risk factors and pre-existing diseases (cancer, angina, stroke, VTE, diabetes, treated hypertension, heavy smoking) generally similar across groups  Idiopathic VTE: excluded pregnancy, delivery, trauma, immobilization, long travel, surgery, and chemotherapy | Large cohort; Prospective design  VTE diagnoses reviewed by medical experts blinded to COC status; VTE diagnoses followed up with information from physician  Low loss to follow-up (1.9 %) and similar between groups  Study powered to detect two-fold difference in risk; Conducted intention-to-treat and as-treated analyses, results did not differ | >20% rate of switching in each COC group;  Percentage of obese and pre-existing arrhythmia higher in DRSP group than LNG group | II-2, good |
| Dinger et al, 2014 [3]  Bayer AG  United States  Austria  Croatia  Germany  Italy  Poland  Sweden | Prospective cohort  United States: 2005-2011  Europe: 2008-2013 | 85,109 women analyzed 206,296 WY observation | Exposure:  Questionnaire  Main outcome:  DVT or PE  Methods to confirm VTE:  Patient questionnaires, verified by MDs | DRSP  LNG | \| **COC** \| **PE** \| **DVT** \| **WY (as treated)** \| **WY (intention to treat)** \| \| --- \| --- \| --- \| --- \| --- \| \| DRSP_24_ \| 1 \| 9 \| 26491 \| 38772 \| \| DRSP_21_ \| 0 \| 8 \| 17112 \| 23721 \| \| LNG \| 3 \| 6 \| 19472 \| 25066 \|  \| **COC** \| **Incidence**  **(VTE/10,000 WY)** \| **Crude HR (95% CI)** \| **Adjusted HR**  **(95% CI)** \| \| --- \| --- \| --- \| --- \| \| *Confirmed VTE* \| \| \| \| \| DRSP_24_ \| 7.2 (4.3-11.2) \| 0.8 (0.5-1.6) \| 0.8 (0.4-1.6) \| \| LNG \| 9.8 5.9-15.2) \| Ref \| Ref \| \| ‘*Idiopathic’ VTE* \| \| \| \| \| DRSP_24_ \| 4.9 (2.6-8.4) \| 0.7 (0.3-1.6) \| 0.7 (0.3-1.6) \| \| LNG \| 7.2 (3.9-12.1) \| Ref \| Ref \|  \| **Other Comparison groups** \| **Crude HR (95% CI)** \| **Adjusted HR**  **(95% CI)** \| \| --- \| --- \| --- \| \| 30mcg EE + DRSP vs. 30mcg EE + LNG \| 1.0 (0.4-2.3) \| 0.9 (0.4-2.1) \| \| 20mcg EE + DRSP vs. 20mcg EE + LNG \| 0.8 (0.3-1.8) \| 0.7 (0.3-1.8) \| | Included starters, switchers or restarters of COC across sites  Reported similar baseline characteristics (age, weight, BMI, treated hypertension, high cholesterol, family history of ATE or VTE, smoking status, diabetes, MI, stroke, cancer, any surgery, depression) across groups  Adjusted HR: age, BMI, current duration of use, and family history of VTE  Idiopathic VTE: excluded pregnancy, delivery, trauma, immobilization, long travel, surgery, and chemotherapy | Large, population-based international cohort  VTE diagnoses reviewed by medical experts blinded to COC status; VTE diagnoses followed up with information from physician  Low loss to follow-up (3.3%) and similar between groups and across US (3.8%) and European (2.6%) sites | No reporting on specific progestogen-containing COC formulations other than DRSP and LNG  Did not account for history of VTE | II-2, good |
| Herings et al., 1999 [24]  Funding not stated  The Netherlands | Cohort  1986-1995 | All female residents in 8 Dutch cities (N =450,000) | Exposure:  Information from PHARMO system (hospital admissions and drug dispensing data)  Main outcome:  VTE (not further specified)  Methods to confirm VTE:  Diagnostic codes from hospital discharges | GTD  LNG DSG | All COCs with 30 mcg EE   \| **COC** \| **VTE** \| **WY** \| **Crude risk/10000 WY** \| **Adjusted RR (95% CI)** \| \| --- \| --- \| --- \| --- \| --- \| \| LNG \| 6 \| 24953 \| Ref \| Ref \| \| DSG \| 19 \| 22373 \| 3.5 (1.4-8.8) \| 4.2(1.7-10.6) \| \| DSG \| 3 \| 2632 \| 4.7 (1.2-19.1) \| 4.5 (1.1-18.2) \| \| GTD \| 5 \| 4982 \| 4.2 (1.3-13.7) \| 3.9 (1.2-12.9) \| | Included all women, ages 15 to 49 with ever use of COC between 1986-1995 per prescription data  Included only first episodes of exclusive use of second or third generation COC  Excluded women with history of VTE, use of anticoagulation, depot hormone drugs, cardiovascular medication or the ‘morning after pill’ and any woman with hospitalization for any reason within 2 months prior to start of COC  Adjusted for year and age | Drug dispensing records from pharmacy files linked to nationwide hospital discharge records with sensitivity and specificity > 95%  Did not validate VTE diagnosis codes with record review for evidence of diagnostic tests or therapeutic interventions | Prescription data may not accurately reflect COC use at time of event  Did not adjust for smoking, BMI, current or recent pregnancy | II-2, poor |
| Lidegaard et al., 2011 [31]  Bayer Schering Pharma  Denmark | Retrospective cohort  2001-2009 | 1296120 women contributing 8010290 women-years of observation | Exposure:  Prescription data from national registry  Outcome: Hospital discharge diagnoses and death registry for fatal events linked to minimum 4 weeks treatment with anticoagulation  Linkages of four national databases | LNG  NGM  DSG  GTD  DRSP  CYP | \| **COC** \| **Confirmed VTE** \| **WY** \| **Adjusted RR (95% CI)** \| \| --- \| --- \| --- \| --- \| \| 30-40 mcg EE \|  \|  \|  \| \| LNG All \| 57 \| 104251 \| Ref \| \| NGM \| 119 \| 267664 \| 1.2 (0.9-1.6) \| \| DSG \| 168 \| 170249 \| 2.2 (1.7-3.0) \| \| GTD \| 575 \| 668355 \| 2.1 (1.6-2.8) \| \| DRSP \| 196 \| 286859 \| 2.1 (1.6-2.8) \| \| CYP \| 88 \| 120934 \| 2.1 (1.5-3.0) \| \| 20 mcg EE \|  \|  \|  \| \| DSG \| 246 \| 470982 \| 1.6 (1.2-2.1) \| \| GTD \| 240 \| 472118 \| 1.7 (1.3-2.3) \| \| DRSP \| 16 \| 23055 \| 2.2 (1.3-3.9) \| | Included all Danish women, ages 15 to 49 identified during the study period  Excluded women with any type of arterial or thrombotic event before the study period, cancer, bilateral salpingo-oophorectomy, hysterectomy, or sterilization, known coagulation defect  Censored women at time of pregnancy, postabortion/post-partum, and at time of major surgery or sugery on lower extremities, and during infertility treatments  Adjusted RRs for age, calendar year, level of education and length of use | Large, population-based study  Validated subset of hospital records with two independent clinical experts blinded to COC to confirm cases; 74% of 200 reviewed charts met 2 of three criteria: positive diagnosis, diagnostic confirmation, minimum of four weeks of anticoagulation | Prescription data may not accurately reflect COC use at time of event  Did not include information about smoking; although report inverse correlation between smoking and education  67.1% of all cases with evidence for anticoagulation treatment, but varied (64-84%) according to progestogen | II-2, fair |
| Ziller et al., 2014 [17]  Funding not stated  Germany | Retrospective cohort  2005-2010 | 1,740,615 women who visited gynecologic practices  68,168 women ages 16-45 with COC prescription included in analyses | Exposure: prescriptions from IMS HEALTH electronic records  Main outcome:  VTE (not further defined)  Methods to confirm VTE:  Diagnosis codes, included only cases which were confirmed by physician (personal communication K. Kostev) | DSG  DRSP  DEG  CMA  CYP  NGM  LNG | \| **COC** \| **% VTE (95% CI)** \| **Adjusted OR (95% CI)** \| \| --- \| --- \| --- \| \| LNG (n=13222) \| 0.03% (0.00-0.06) \| Ref \| \| CYP (n=5837) \| 0.03% (0.00-0.08) \| 0.66 (0.07-5.89) \| \| DRSP (n=15572) \| 0.04% (0.01-0.08) \| 1.57 (0.46-5.38) \| \| DSG (n=8375) \| 0.06% (0.01-0.11) \| 1.95 (0.52-7.29) \| \| DNG (n=13785) \| 0.09% (0.04-0.14) \| 2.97 (0.96-9.24) \| \| NGM (n=2183) \| 0.09% (0.00-0.22) \| 3.24 (0.59-17.75) \| | Included women, ages 16 to 45 years with prescription and receiving follow up at least one year after initial prescription  Excluded history thrombosis, anticoagulant prescription  Adjusted for age, BMI, insurance, region, history hormonal use, history other sex hormone use, recent surgery, pregnancy, heart disease |  | Small number of VTE events (38 overall, did not report number in each COC group)  VTE outcomes not verified by medical record review  Did not state whether VTE diagnoses required anticoagulant prescription  Only looked back 1 year for any COC use  Report risk per user rather than per person-year of exposure to account for periods of non-use | II-2, poor |

Abbreviations: ATE, arterial thromboembolism; BMI, body mass index; CI, confidence interval; COC, combined oral contraceptive; CYP, cyproterone acetate; DRSP, drospirenone; DSG, desogestrel; DVT, deep venous thrombosis; EE, ethinyl estradiol; GTD, gestodene; HR, hazard ratio; LNG, levonorgestrel; MI, myocardial infarction; NGM, norgestimate; NR, not reported; PE, pulmonary embolism; RR, relative risk; VTE, venous thromboembolism; WY, woman-year.
